# Antigenic and 3D structural characterization of soluble X4 and hybrid X4-R5 HIV-1 Env trimers

**DOI:** 10.1186/1742-4690-11-42

**Published:** 2014-05-30

**Authors:** Philipp Arnold, Patricia Himmels, Svenja Weiß, Tim-Michael Decker, Jürgen Markl, Volker Gatterdam, Robert Tampé, Patrick Bartholomäus, Ursula Dietrich, Ralf Dürr

**Affiliations:** 1 Institute of Zoology, Johannes Gutenberg University, Mainz, Germany; 2 Molecular Virology, Georg-Speyer-Haus, Institute for Tumor Biology and Experimental Therapy, Paul-Ehrlich-Str. 42-44, 60596 Frankfurt, Germany; 3 Institute of Biochemistry, Goethe University, Frankfurt, Germany; 4 Current address: Anatomical Institute, Christian-Albrecht’s University, Kiel, Germany; 5 Current address: Biochemistry Center (BZH), Heidelberg University, Heidelberg, Germany; 6 Current address: Department of Molecular Epigenetics, Helmholtz Center Munich, Center for Integrated Protein Science Munich (CIPSM), Munich, Germany; 7 Current address: Institute for Experimental Infection Research, Twincore, Center for Experimental and Clinical Infection Research, Hannover, Germany; 8 Current address: Department of Pathology, New York University, School of Medicine, New York City, USA

**Keywords:** HIV-1, Soluble gp140 Env, CXCR4, CCR5, Tropism, V3 loop, 3D EM, Single particle analysis, CD4 binding site, Open structure

## Abstract

**Background:**

HIV-1 is decorated with trimeric glycoprotein spikes that enable infection by engaging CD4 and a chemokine coreceptor, either CCR5 or CXCR4. The variable loop 3 (V3) of the HIV-1 envelope protein (Env) is the main determinant for coreceptor usage. The predominant CCR5 using (R5) HIV-1 Env has been intensively studied in function and structure, whereas the trimeric architecture of the less frequent, but more cytopathic CXCR4 using (X4) HIV-1 Env is largely unknown, as are the consequences of sequence changes in and near V3 on antigenicity and trimeric Env structure.

**Results:**

Soluble trimeric gp140 Env constructs were used as immunogenic mimics of the native spikes to analyze their antigenic properties in the context of their overall 3D structure. We generated soluble, uncleaved, gp140 trimers from a prototypic T-cell line-adapted (TCLA) X4 HIV-1 strain (NL4-3) and a hybrid (NL4-3/ADA), in which the V3 spanning region was substituted with that from the primary R5 isolate ADA. Compared to an ADA (R5) gp140, the NL4-3 (X4) construct revealed an overall higher antibody accessibility, which was most pronounced for the CD4 binding site (CD4bs), but also observed for mAbs against CD4 induced (CD4i) epitopes and gp41 mAbs. V3 mAbs showed significant binding differences to the three constructs, which were refined by SPR analysis. Of interest, the NL4-3/ADA construct with the hybrid NL4-3/ADA CD4bs showed impaired CD4 and CD4bs mAb reactivity despite the presence of the essential elements of the CD4bs epitope. We obtained 3D reconstructions of the NL4-3 and the NL4-3/ADA gp140 trimers via electron microscopy and single particle analysis, which indicates that both constructs inherit a propeller-like architecture. The first 3D reconstruction of an Env construct from an X4 TCLA HIV-1 strain reveals an open conformation, in contrast to recently published more closed structures from R5 Env. Exchanging the X4 V3 spanning region for that of R5 ADA did not alter the open Env architecture as deduced from its very similar 3D reconstruction.

**Conclusions:**

3D EM analysis showed an apparent open trimer configuration of X4 NL4-3 gp140 that is not modified by exchanging the V3 spanning region for R5 ADA.

## Background

The envelope glycoproteins (Env) of HIV are the key elements mediating viral entry and represent the major target for HIV-neutralizing antibodies [1,2]. Env derives from gp160 precursors that trimerize in the endoplasmic reticulum and, following cleavage in the Golgi, generate non-covalently associated trimers of gp120 and gp41 heterodimers. CD4-gp120 binding induces conformational changes in Env, both to expose epitopes for subsequent interaction with coreceptors (CCR5 or CXCR4) and to activate the gp41 transmembrane subunits for membrane fusion [3].

According to their coreceptor usage, HIV-1 strains can be subdivided into CCR5- (R5) and CXCR4-tropic (X4) variants. While R5 strains are usually transmitted and predominate at early stages of infection, X4 variants are found in approximately 50% of subtype B infected patients in chronic stages of the disease and coincide with a rapid CD4 T-cell loss and an accelerated progression to AIDS (see reviews [4,5]). Since the emergence of X4 variants correlates with a worse prognosis and excludes the use of CCR5 inhibitors in patients, a better characterization of X4 HIV-1 is an urgent need. CXCR4 usage is mainly mediated by mutations in the variable loop 3 (V3) of gp120, especially at the V3 stem, providing an increased positive net charge [6-8]. In addition to single mutations in V3, conserved secondary structural constraints have been shown to contribute to coreceptor choice [9,10]. Besides coreceptor selection and interaction, V3′s conserved structural constraints render it into one of four Env regions able to induce cross-clade neutralizing antibodies [11-14].

Soluble trimeric envelope proteins (gp140), composed of gp120 linked to the extracellular part of gp41, are useful tools in both immunological and structural studies [15,16]. Expression of gp140 can be achieved either by deleting the internal protease cleavage site [17] or by introduction of an intraprotomeric stabilizing disulfide bridge (gp140 SOSIP) [18,19]. Recently, efforts were undertaken to determine the trimeric Env structure of membrane solubilized or recombinantly expressed gp140 constructs, either in the uncleaved precursor state [20-22] or in the more mature cleaved state in gp140 SOSIPs [23-32]. Subramaniam and colleagues demonstrated that soluble gp140 SOSIP constructs display an almost identical gp120 molecular arrangement as that observed in intact HIV-1 virions, both in the unliganded and the CD4 activated state [24]. Mutational analysis of trimeric Env with deletions of certain variable loops yielded important information on their location and influence on trimer stability [25,29].

All the known structural studies so far have characterized trimeric Env, either from intact virions or from soluble gp140 constructs derived from R5 HIV-1 or from SIV. This is complemented by the recent X-ray structures of the cellular receptors CXCR4 and CCR5, which interact with Env and thereby induce fusion [33,34]. However, no quaternary structural data is available so far for X4 HIV-1 Envs. In the present study, we aimed at characterizing the antigenic and structural characteristics of trimeric uncleaved gp140 from the prototypic T-cell line-adapted X4 subtype B strain NL4-3 [35]. Additionally, a mutant construct was generated, where a V3 spanning region was exchanged for that of the primary R5 strain ADA (NL4-3/ADA). The exposed V3 is embedded between the immunosilent C2 and C3 regions that also contain elements from the discontinous CD4 binding site (CD4bs). The exchange of V3 with adjacent small elements of C2 and C3 should support the display of the exchanged V3 in a conformational context and highlight the effects of a combined CD4 binding site originating from two different HIV-1 strains. Our study aimed at (1) giving first insights into the quaternary structure of an X4 Env and (2) address consequences of introducing an extended R5 V3 region into X4 NL4-3 gp140 on antigenicity and structure.

## Results

### Recombinant expression and characterization of NL4-3 and NL4-3/ADA gp140 trimers

**Figure 1:**
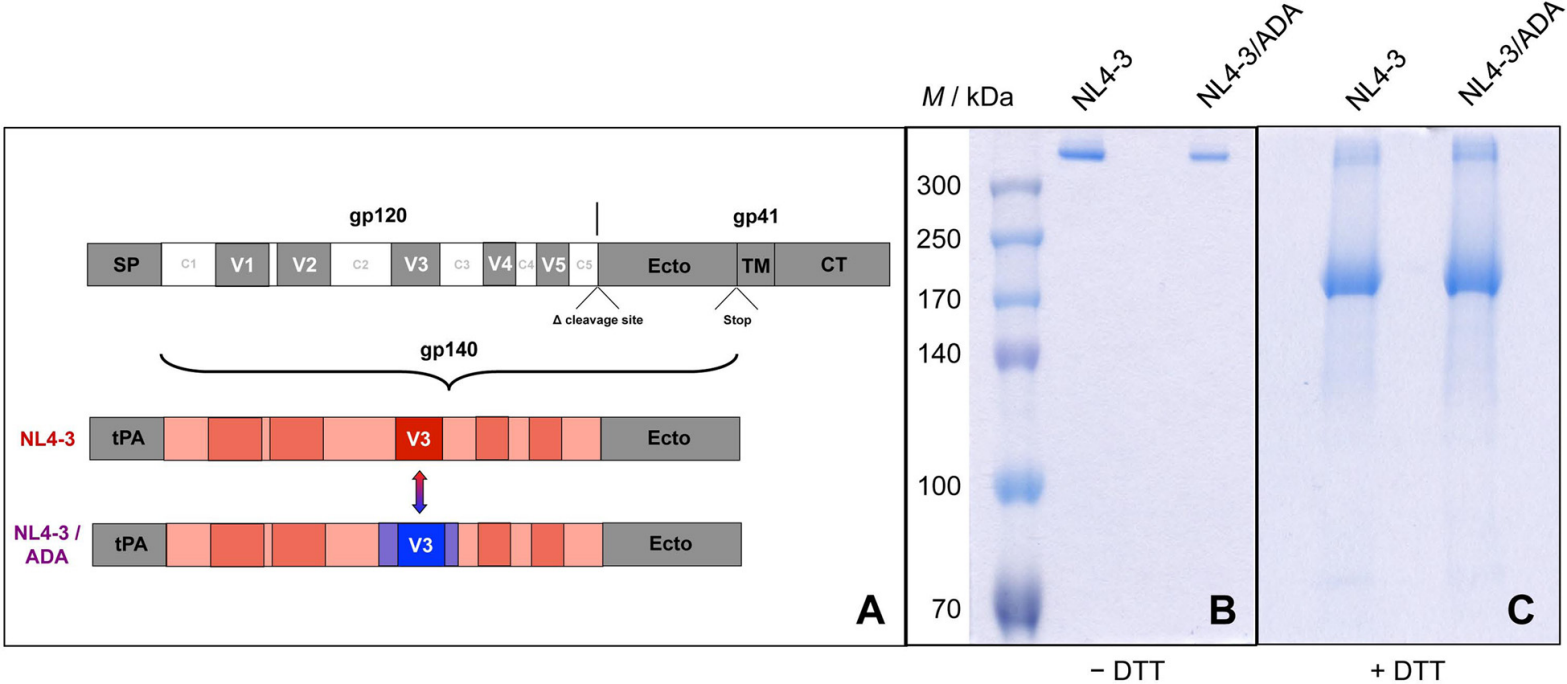
**Scheme of the NL4-3 and NL4-3/ADA constructs and protein analysis by SDS-PAGE. (A)** Scheme of gp140 expression constructs indicating cloning of the HIV-1 NL4-3 Env ectodomain with tPA leader sequence and deleted cleavage site. The hybrid construct NL4-3/ADA was generated by exchanging the V3 domain in conjunction with adjacent constant regions of the X4 tropic NL4-3 construct with the respective sequences of the R5 tropic HIV-1 ADA strain. The exchange of the extended V3 region contains two short linear elements of the discontinuous CD4 binding site (see also Additional file 1 and 2). **(B**, **C)** SDS-PAGE analysis of purified NL4-3 and NL4-3/ADA gp140 trimers under non-reducing **(B)** and reducing conditions (+50 mM DTT) **(C)**.

Two uncleaved gp140 constructs were generated, NL4-3 gp140 derived from the X4 prototypic HIV-1 subtype B strain NL4-3 and a hybrid mutant (NL4-3/ADA) with a V3 spanning region exchanged for that of the R5 subtype B strain ADA (Figure 1A). The exchanged region comprises the complete V3 loop as well as adjacent elements of C2 (45 aa) and C3 (14 aa). This strategy enables V3 presentation in its parental context, preserves all N-glycosylation sites as well as adjacent CD4bs elements, whereas the exchange of threonine for asparagine at position 277 marginally affects the epitopes of some CD4bs mAbs (see sequence alignment and epitopes in Additional files 1 and 2). The recombinantly expressed gp140 proteins were purified with at least two sequential purification steps to obtain pure gp140 trimers devoid of monomers and dimers (Figure 1B, Additional file 3A,B). Gp140 trimers are composed of gp140 monomers that migrate around 180 kDa upon DTT treatment in SDS-PAGE (Figure 1C). The uncleaved R5 ADA gp140 construct was biochemically analyzed in detail [17], and was also highly purified in its trimeric form for comparison (see Additional file 3C).

### Antibody binding studies by ELISA and SPR

**Figure 2:**
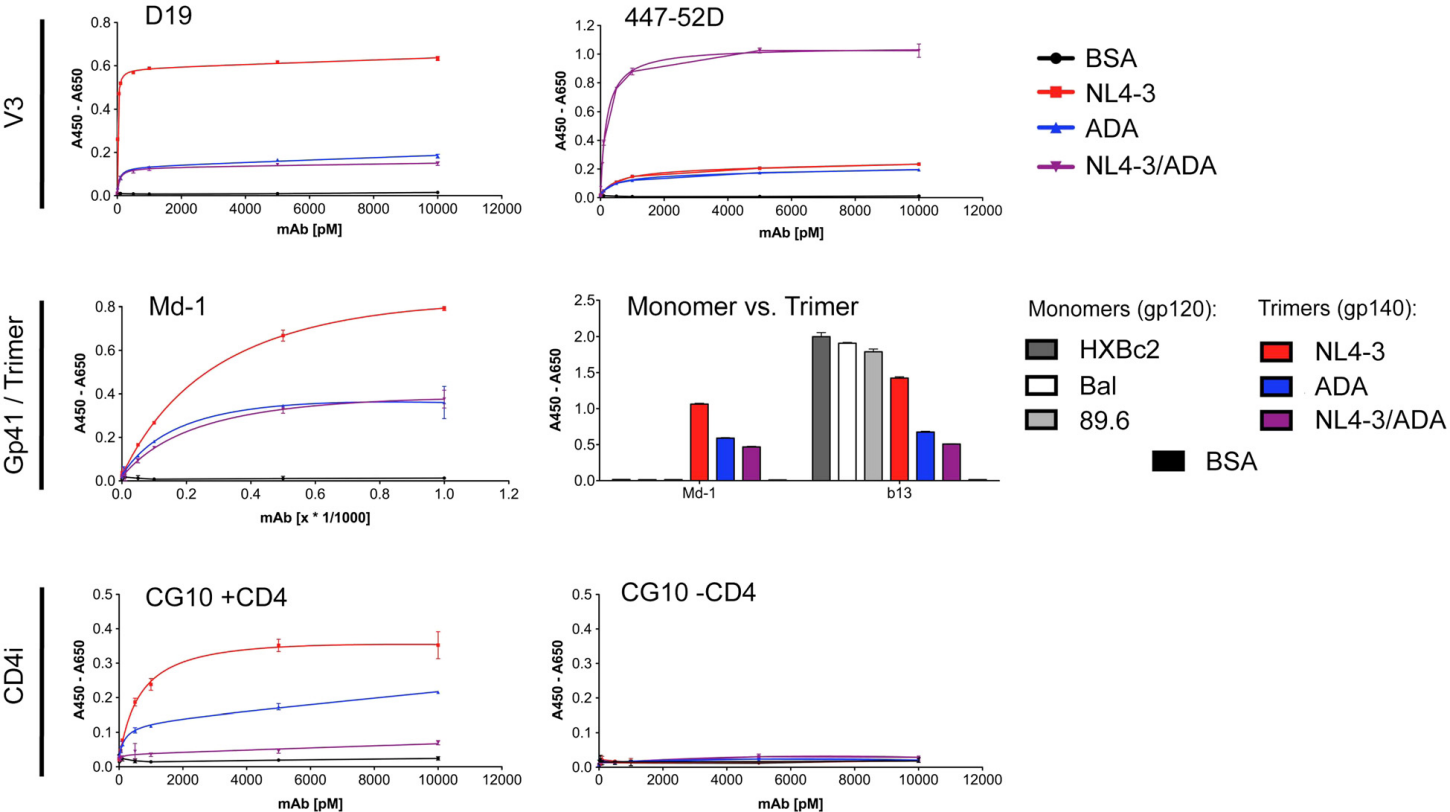
**Antibody binding to gp140 constructs in ELISA experiments.** The antigenic profiles of the gp140 constructs NL4-3 (X4), ADA (R5) and the hybrid NL4-3/ADA were evaluated in ELISA experiments with selected monoclonal antibodies (mAbs) against the gp120 V3 loop (447-52D and D19) [36,37] and the gp41 region (Md-1) [38]. Functional exposure of coreceptor binding epitopes upon CD4 activation was monitored with strictly CD4 dependent mAb CG10 [39]. Where applicable, nonlinear regression fits are shown in the diagrams instead of connected data points. Derived K_D_ values (PRISM software) are listed in the table of Additional file 5. The purified gp140 timers (NL4-3, ADA and NL4-3/ADA) were compared with gp120 monomers (X4 HXBc2, R5 Bal, R5X4 89.6; Immune Technology) regarding their reactivity with CD4bs mAb b13 (5 nM) and trimer specific gp41 mAb Md-1 (1:2000 dilution). The data is representative for at least three independent replicate experiments with each data point of the binding curves determined in triplicates with indicated mAb concentrations. Error bars indicate the standard deviation.

**Figure 3:**
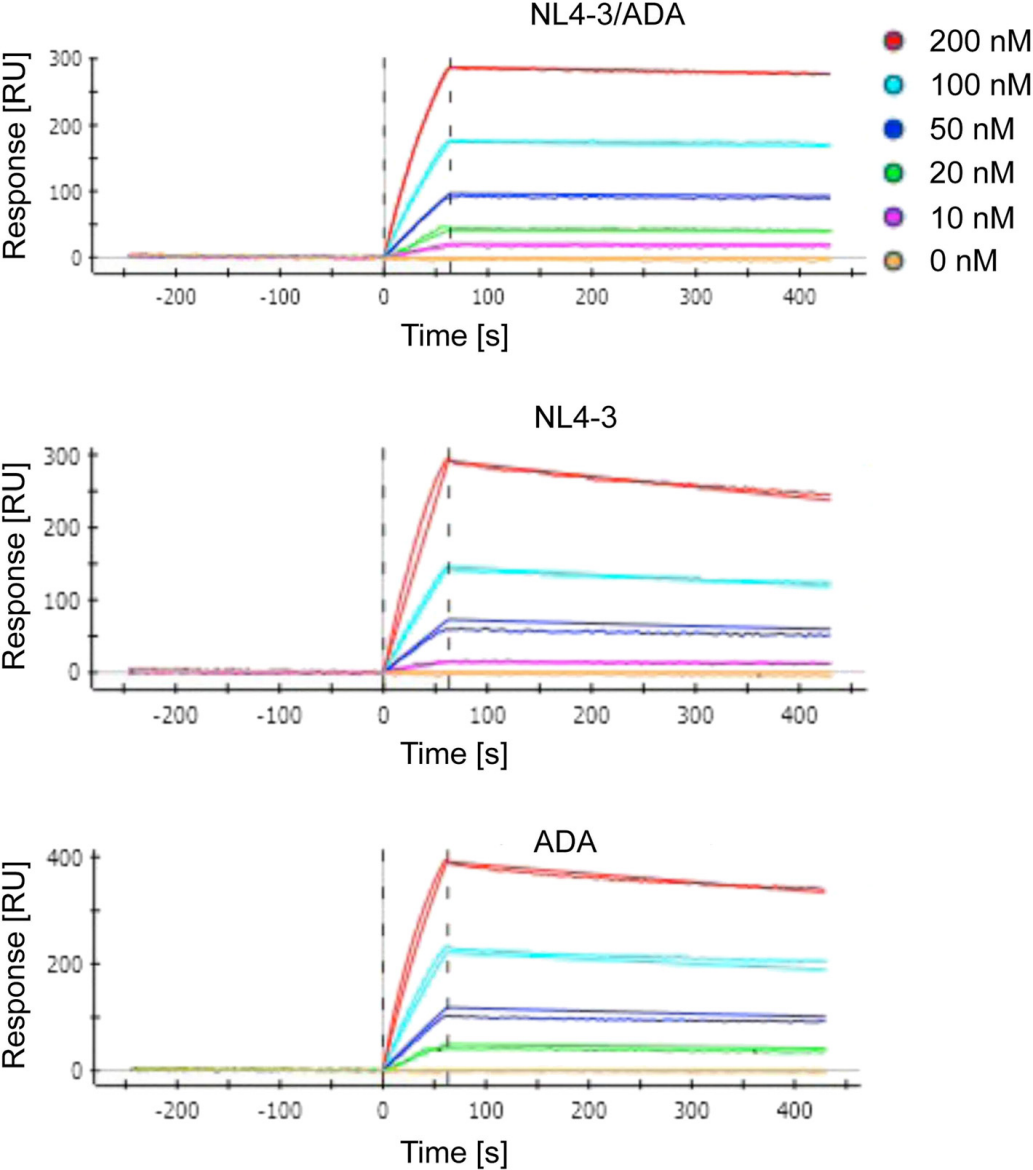
**Kinetics of 447-52D antibody binding to gp140 constructs with SPR measurements.** ProteOn XPR36 measurements were performed with immobilized 447-52D antibody (ligand) and rising concentrations of the different gp140 constructs (analyte). Note the remarkable slower dissociation of the NL4-3/ADA construct from the 447-52D mAb in contrast to the NL4-3 and ADA construct, which is responsible for its higher affinity to 447-52D (see also Figure 2 and table of Additional file 6 with listed K_D_, k_on_ and k_off_ rates).

**Figure 4:**
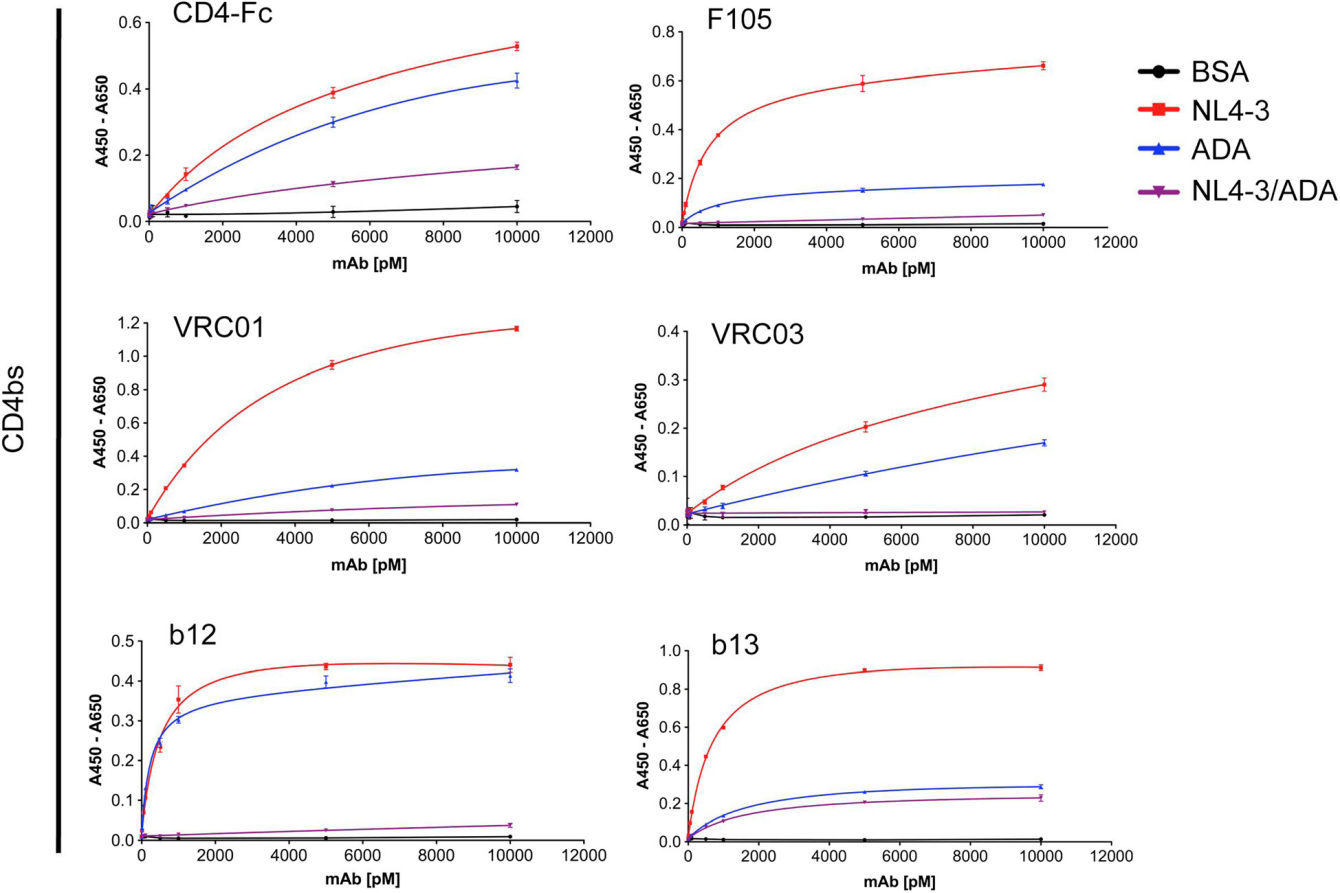
**CD4bs antibody binding to gp140 constructs in ELISA experiments.** The CD4bs reactivity of the gp140 constructs NL4-3 (X4), ADA (R5) and the hybrid NL4-3/ADA was evaluated in ELISA experiments with CD4-Fc and five selected monoclonal antibodies: VRC01, VRC03 (both [40]), b12 [41], b13 [41] and F105 [42]. Where applicable, nonlinear regression fits are shown in the diagrams instead of connected data points. Derived K_D_ values (PRISM software) are listed in the table of Additional file 5. The data is representative for at least three independent replicate experiments with each data point of the binding curves determined in triplicates with indicated mAb concentrations. Error bars indicate the standard deviation.

We studied the antigenic properties of our gp140 constructs by analyzing the reactivity with monoclonal antibodies (mAbs)/antibody constructs directed against gp120 V3 (447-52D, D19), CD4bs (CD4-Fc, VRC01, VRC03, b12, b13, F105), the coreceptor binding site (CG10, 17b) and gp41 (Md-1, 2F5, 246-D) (Figures 2, 3, 4, Additional file 4 and tables in Additional files 5 and 6). The V3 mAbs D19 and 447-52D reacted with all gp140 recombinant proteins proving the exposure of V3 in all constructs. MAb D19 preferentially recognizing V3 from X4 strains [36], showed enhanced reactivity with the X4 NL4-3 gp140 compared to the R5 ADA gp140, as expected. Consequently, the exchange of the X4 V3 region for the R5 ADA V3 reduced mAb D19 binding to levels of the R5 ADA control, as seen by comparable A_max_ (Figure 2) and affinity (see K_D_ values in Additional file 5). In contrast, mAb 447-52D, directed against the tip of V3, showed similar binding curves for NL4-3 and ADA gp140, however A_max_ and affinity for the hybrid NL4-3/ADA construct were remarkably increased. We confirmed this finding by SPR measurements with an inversed binding setup compared to the ELISA experiments, *i.e.* immobilization of mAb 447-52D and administration of the gp140 constructs as analytes (Figure 3 and table in Additional file 6). The k_on_ rates of the different gp140 constructs for mAb 447-52D were comparable, however we observed a much slower dissociation of the hybrid NL4-3/ADA from mAb 447-52D with k_off_ values 5 times lower compared to the other constructs. This resulted in lower K_D_ values and enhanced binding signals in end point analyses. Gp41 antibodies Md-1, 2F5 and 246-D were reactive with all gp140 constructs (Figure 2 and Additional files 4 and 5). The reactivity with the trimer specific antibody Md-1 confirmed the trimeric state of our gp140 constructs (Figure 2). Despite the presence of several antibody epitopes in all gp140 constructs, we detected quantitative differences: in most cases the mAbs showed best binding to NL4-3 gp140, reduced binding to ADA gp140 and strongly reduced binding to NL4-3/ADA. Exceptions are mAbs D19, Md-1 and b13 with comparable binding levels to ADA and NL4-3/ADA and the V3 mAb 447-52D, which binds by far best to the hybrid NL4-3/ADA.

ELISA experiments with the coreceptor binding site antibody CG10, which is strictly CD4 dependent, showed enhanced binding to NL4-3 gp140 compared to ADA gp140 after CD4 activation (Figure 2). Accordingly, the less CD4 dependent CD4i antibody 17b bound preferentially to NL4-3 after CD4 activation, however, similarly bound to both NL4-3 and ADA gp140 without CD4 activation (Additional file 4). The hybrid NL4-3/ADA gp140 exhibited only minimal binding to either CD4i antibody. These findings prompted a thorough analysis of the CD4 binding characteristics of our gp140 constructs. For a detailed CD4bs analysis, we employed a CD4-Fc construct and the five CD4bs mAbs VRC01, VRC03, b12, b13 and F105 (Figure 4 and Additional file 2), which differ in their neutralizing capacity and their steric hindrance in binding to native trimers. NL4-3 gp140 is most reactive with all constructs targeting the CD4bs. In contrast, the NL4-3/ADA hybrid shows massively reduced reactivity of its CD4bs despite the presence of all critical elements of the discontinuous epitope (Additional files 1 and 2). While moderate binding to CD4-Fc and b13 mAb is still observed, the hybrid NL4-3/ADA gp140 almost completely lost its accessibility for the other CD4bs mAbs compared to its parental NL4-3 and ADA gp140 constructs. Although only one amino acid (aa 277 T -> N) is changed within the epitope for some CD4bs mAbs (shown for b12, VRC01 and F105 in Additional file 2), the exchange in the V3 flanking region contains a total of 11 altered amino acids (Additional files 1 and 2). This may result in subtle structural alterations within the conformationally sensitive CD4bs resulting in reduced binding and activation by CD4 and CD4bs mAbs.

### 3D reconstruction of NL4-3 and NL4-3/ADA gp140 trimers

**Figure 5:**
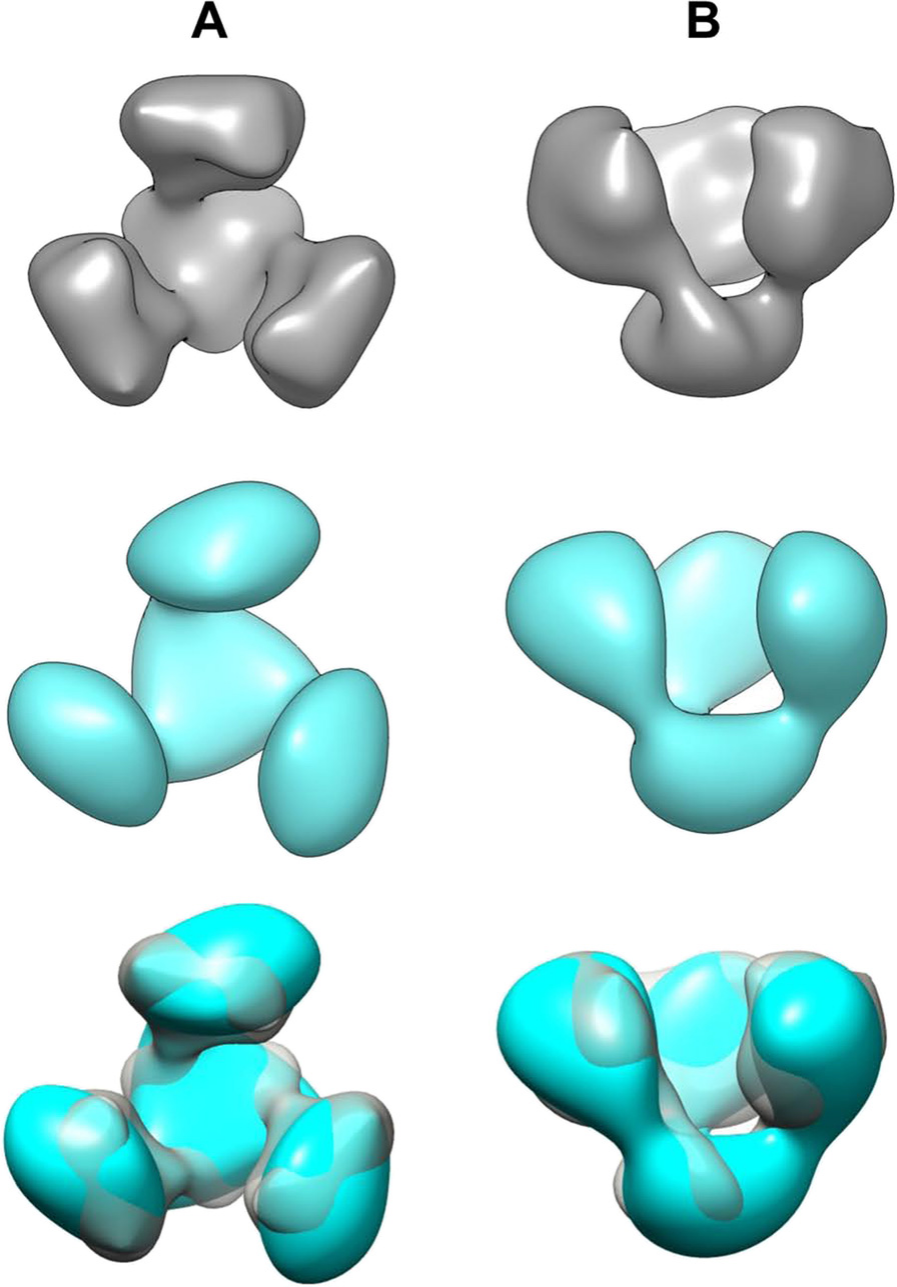
**3D reconstruction of NL4-3 and NL4-3/ADA gp140 trimers.** Density maps of NL4-3 gp140 trimer (grey) and NL4-3/ADA gp140 trimer (cyan) viewed along (**A**, top view) and perpendicularly to its threefold symmetry axis (**B**, side view). Both density maps show a trimeric propeller-like Env architecture with an open conformation. The three masses are only connected in the lower part. The bottom row depicts the superposition of the density maps. Note the high overall congruency; the subtle differences in the tilting of the upper masses and the rotation of the triangular lower mass are not significant at the present resolution of 25 Å.

Using electron microscopy and single particle analysis, we generated 3D reconstructions from trimeric NL4-3 (EMDB 2657 [43]) and NL4-3/ADA gp140 (EMDB 2659 [44]). Gp140 density maps were generated from negatively stained single particles through an iterative procedure of 3D alignment, classification, and averaging using about 3,000 particles per data set (see random selection in Additional file 7). By these means we obtained structural information from trimeric Env derived from an X4 HIV-1 strain and observed for the first time the effects of exchanging the V3 spanning region on the overall trimer structure at the given resolution of ~25 Å (Figure 5; NL4-3 in grey, NL4-3/ADA in cyan). In side view, the structures display an open upper part with three masses that are connected with one bridge each to a compact mass in the lower part (Figure 5B). The upper masses are directed upwards and are almost parallel to the three-fold symmetry axis. Viewed from the top (Figure 5A), the density maps have a propeller-like appearance. The upper masses are located laterally to the central lower mass with its apices tilting sideways and for NL4-3/ADA also slightly outwards. Superposition of both density maps (Figure 5, bottom row) reveals that they clearly resemble each other in their overall architecture. Whether the small differences between both density maps in tilt and rotation angle of the four masses are significant is not clear at the present resolution.

### Docking of unliganded NL4-3 and NL4-3/ADA gp140

**Figure 6:**
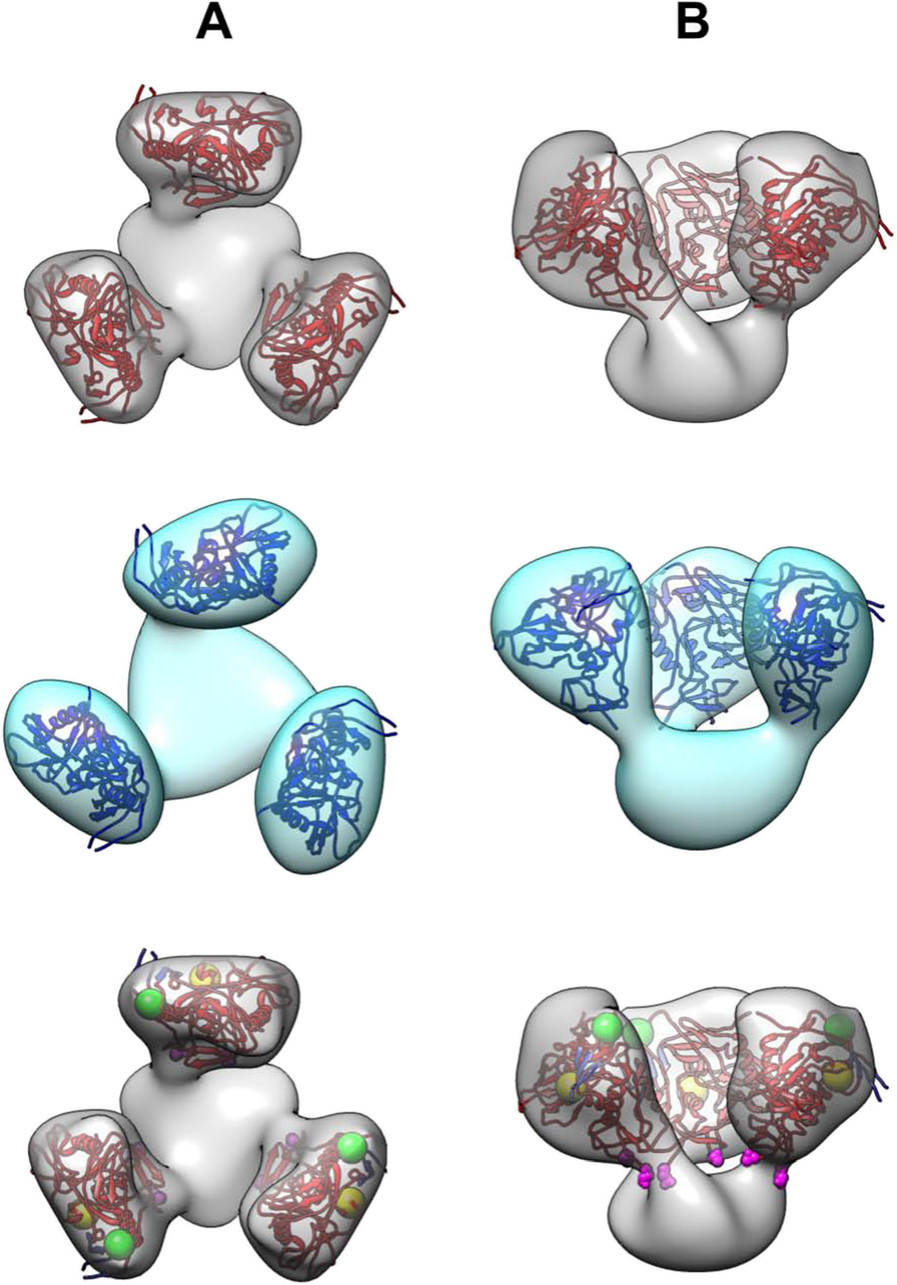
**Fitting of gp120 X-ray structures into NL4-3 and NL4-3/ADA gp140 density maps.** Automated fitting of a gp120 X-ray structure (pdb 2NY7) into NL4-3 (grey) and NL4-3/ADA gp140 density maps (cyan), viewed from top **(A)** and side view **(B)**. In the bottom row, the 3D model of NL4-3 gp140 is shown with the locations of the V3 loop and the CD4 binding site indicated with green and yellow balls, respectively. Magenta balls represent the N- and C-terminal stumps of gp120. The bridging sheets are colored in blue.

At the mass-corrected threshold, the three protruding masses of both 3D reconstructions resembled an unliganded gp120 molecule in size and overall shape (see for example pdb-ID 2NY7 [45]). Copies of the latter were docked as follows (Figure 6): First, the gp120 structures were oriented manually with their N- and C-terminus (magenta spheres in Figure 6 bottom row) directing towards the single larger central mass, which is supposed to represent the ectodomain of gp41. After this pre-orientation, automated rigid-body fitting yielded a stable result, with the N-glycosylation sites (spheres in Additional file 8) and the CD4 binding sites (yellow spheres in Figure 6 bottom row) pointing outwards. Significantly, with these sites pointing inwards, stable automated docking was not achieved. This result was obtained with both 3D reconstructions. In both cases, the only stable alternative was with the N- and C-termini pointing 180° away, *i.e.* opposite to the supposed ectodomain, which can be excluded for structural reasons. The allocation of the V3 loops and the bridging sheet are indicated with green spheres and blue beta sheets, respectively (Figure 6 bottom row and its animated version in Additional file 9). Automated fitting of both 3D reconstructions revealed that their docked X-ray structures were almost superimposed (Additional file 10). This underlines their similarity, as the slight differences are within the resolution limit.

## Discussion

In this study, the prototypic X4 NL4-3 TCLA strain [46] was used to derive soluble uncleaved gp140 constructs with the parental X4 and a recombinant R5 ADA V3 spanning region for antigenic and structural analyses. V3 exchanges between R5 and X4 HIV-1 were performed previously and proven to be sufficient to mediate a change in cell tropism [6,47-49]. An increase in positive net charge mediated by only a few amino acid changes, mainly at positions 11, 24 or 25 of the V3 loop, were described as being sufficient to shift binding from CCR5 to the more negatively charged CXCR4 coreceptor [50]. The V3 exchange in our construct resulted in a decrease in net charge from +9 in the X4 NL4-3 V3 loop (9 positively R/K/H residues vs. no negatively charged D/E residues) to +5 in the R5 ADA V3 loop (7 R/K/H vs. 2 D/E) including an exchange at position 25 from lysine (K) to aspartic acid (D). As expected, mAb D19, which preferentially binds X4 variants [36], strongly bound to the X4 NL4-3 gp140 construct and the V3 exchange to R5 ADA reduced D19 binding to levels of the paternal R5 ADA gp140 according to the expected acquisition of R5 like properties.

3D reconstructions of both constructs were generated from purified Env trimers. Thus, the reconstruction of the complexes was straight forward and no *in silico* separation of particles had to be done. As both data sets were split prior to the reconstruction process noise refinement was minimized. Although at the present resolution of ~25 Å structural details still remain obscure, the overall quaternary architecture can be deduced. The propeller-like trimer configurations with a common basal stalk are similar in both constructs and furthermore are congruent to recently published structures of soluble HIV-1 Env (*e.g.*[24,29]) and a CD4 independent SIV Env ([51]; see also Additional file 11). Automated fitting of the gp120 X-ray structure [45] resulted in a reasonable location of reference epitopes like the CD4 binding site, V3 loop, coreceptor binding or N-glycosylation sites (Figure 6 and Additional file 8).

The gp140 construct from the TCLA X4 strain NL4-3 shows a marked open configuration, which resembles the open architecture of CD4 independent SIV Env [51,52] but differs considerably from published R5 trimer structures derived from HIV-1 subtype B [20,21,24,53,54] (see Additional files 11 and 12). These subtype B R5 structures share a closed Env configuration with a tight assembly of the three gp120 protomers at the top. The closed R5 conformation was observed for membrane-associated Env on virions as well as for soluble SOSIP gp140 constructs, independently of their origin from laboratory-adapted (Bal) or primary (JRFL) strains. Recently, high resolution Env structures were achieved from stabilized SOSIP gp140 trimers derived from HIV-1 subtype A strains that are known as mainly CCR5 tropic [26,30,35]. These structures also feature a closed conformation at a much higher resolution.

The X4 gp140 construct in this study is derived from the laboratory-adapted strain NL4-3 and therefore, the structure may not reflect the general structure of native Env from primary X4 strains. Alternatively, the open structure may rather reflect structural consequences of lab adaptation. However, the side-by-side comparison of our X4 NL4-3 and an R5 Bal construct, which are both derived from laboratory-adapted HIV-1 strains ([53,54], Additional file 11) reveal marked differences in the “openness” of the trimers. Thus, despite possible adaptations during *in vitro* culture eventually resulting in a more opened Env structure, still X4 Env has a more open architecture than R5 Env. Therefore, the open structure of our X4 NL4-3 Env seems to be primarily due to the X4 phenotype or to inherent features of our uncleaved gp140 immunogens, rather than to culture adaptation. Future studies are needed to confirm these findings with more mature cleaved Env derived from primary X4 strains.

It has been known for long time that TCLA viruses are more sensitive to antibody neutralization than primary strains and it was assumed that this correlates with better accessibility of critical antibody epitopes [55]. However, by using genetically related primary and TCLA viruses, it was shown that their differential neutralization profiles are not related to differences in antibody accessibility and binding but rather result from differences in subsequent entry processes [56]. SHIV macaque models of HIV-1 suggest that the evolution of X4 viruses *in vivo* requires an opening of Env as an early event during coreceptor switch that mediates better access to CD4 and moreover an increased neutralization sensitivity by CD4bs antibodies [57,58]. Accordingly, our ELISA analyses revealed an enhanced accessibility of the CD4bs from the X4 NL4-3 construct in comparison to R5 ADA (Figure 4). We consistently observed higher binding of the five CD4bs mAbs VRC01, VRC03, b12, b13 and F105 as well as CD4-Fc to NL4-3 gp140. Additionally, both coreceptor binding site antibodies, 17b (Additional file 4) and CG10 (Figure 2), the latter being strictly CD4 dependent, exhibited an increased maximum binding capacity for NL4-3 gp140 compared to ADA gp140 upon CD4 activation. Notably, the less CD4 dependent mAb 17b, which even can induce the expression of the coreceptor binding sites in the absence of CD4 in combination with an opening of the trimer structure [32], does not exhibit preferential binding to NL4-3 gp140 without prior CD4 activation. Thus, better antibody access to X4 NL4-3 gp140 is mainly observed for mAbs involving the CD4bs, however this is also true for the gp41 mAbs tested in this study including the trimer specific mAb Md-1.

The NL4-3/ADA hybrid shows considerable reactivity with V3 and gp41 antibodies, which is comparable to that of the ADA construct for mAb D19 and Md-1. Surprisingly, the exchange of the V3 region resulted in substantially enhanced binding of mAb 447-52D, directed against the tip of V3. Similar k_on_ rates in combination with significantly decreased k_off_ rates (Figure 3 and Additional file 6) are indicative of structural constraints impairing antibody dissociation from the hybrid NL4-3/ADA construct resulting in increased overall binding by ELISA (Figure 2). There is also binding of the hybrid construct to CD4 (CD4-Fc), although strongly reduced with respect to ADA and NL4-3 Env (Figure 4). This is remarkable, as the CD4bs core epitope in the mutant construct is identical to that of the NL4-3 construct and there is only one exchange of a threonine to asparagine at position 277 affecting some CD4bs mAb epitopes (see Additional file 2). According to the presence or absence of aa 277 in the respective CD4bs mAb epitopes, we observe different degrees of CD4bs mAb binding ranging from very weak/absent binding (*e.g.* b12, F105, VRC01) to binding comparable to ADA gp140 (b13). Of note, either amino acid at this position works for efficient CD4 and CD4bs mAb binding in their parental NL4-3 or ADA strains. Therefore, it seems likely that our “artificial” combination of two themselves functional CD4 binding sites (NL4-3 and ADA) leads to an altered conformational arrangement of the epitope that limits CD4/CD4bs antibody binding or accessibility [32,59,60]. Consequently, also binding of CD4i mAbs to NL4-3/ADA is drastically reduced (CG10) or absent (17b).

Although this impaired CD4 activation capacity of the NL4-3/ADA hybrid does not allow us to draw any functional conclusions or to address the degree of “openness” by correlations to CD4bs antibody reactivities, it is remarkable that the exchange of the V3 spanning region of X4 NL4-3 for that of R5 ADA did not abrogate the open conformation of the NL4-3 Env. Despite the fact that the V3 exchange resulted in the expected reduced binding of mAb D19 in the NL4-3/ADA hybrid comparable to that of R5 ADA, the overall structure still resembled the original NL4-3 trimer. Thus, multiple mutations at different sites in and outside the NL4-3 V3 may contribute to the open conformation of the NL4-3 gp140 construct, which might partially reflect the documented adaptations of X4 viruses during the process of coreceptor switch [61,62].

## Conclusions

In our study, the soluble NL4-3 HIV-1 gp140 construct served as an immunogenic mimic of a prototypic T-cell line-adapted X4 strain. This is the first 3D reconstruction of an X4 gp140 trimer. It displayed a remarkably open propeller-like structure, contrasting with recent more closed R5 HIV-1 Env structures. Structural studies with higher resolution using either soluble cleaved trimeric Env mimetics or virus bound native Env spikes from different primary X4 strains are needed to determine if the open conformation is a common feature of X4 viruses. Structural studies with recombinant Env trimers combined with functional studies of the respective viruses would further help to elucidate the contribution of different Env regions with regard to coreceptor choice and cell culture adaptation.

## Methods

### Cloning of gp140 constructs

ADA gp140 was cloned as described previously [17]. For 2F5 binding analyses, an ADA gp140 construct was used with extended 19 aa at its C-terminus (encoding for the complete MPER region) and additional 6× His tag. Cloning of NL4-3-gp140 was performed similarly, including the complete MPER region at the C-terminus. For details see Additional file 13.

### Recombinant expression and purification of gp140

Recombinant expression was performed in CHO-Lec3.2.8.1 cells for ADA gp140 [17]. NL4-3 gp140 was expressed similarly in CHO-K1 cells (Lonza) or CHO-Lec1 cells (ATCC) with no effects on structural outcome. Stably transfected cells were induced for expression with 4 mM sodium butyrate and supernatants were harvested after six days. Genomic DNA preparations from the stably gp140 expressing cell lines confirmed the integrity of the expression constructs. In addition to stable expression of gp140 proteins, transient expressions were performed (CHO-K1 cells, Lonza) that were similar in outcome concerning biochemical analysis but superior for structural analysis. Supernatants of transient expressions were harvested two days after transfection. For purification of gp140 trimers, sequential purification steps were performed beginning with *Galanthus nivalis* lectin (Sigma) batch purification and elution with 0.5 M Methyl-α-D-mannopyranoside. The second purification step consisted of size-exclusion chromatography or glycerol gradient centrifugation. For size exclusion chromatography we run a HiLoad™ 16/60 Superdex™ 200 prep grade column (GE Healthcare) at 0.4 ml/min with 0.5 fraction size on an Äkta chromatography system. Glycerol gradient centrifugations were performed with a 10 - 30% glycerol gradient and a 5% glycerol cushion in 4 ml Beckman Polyallomer UZ tubes in a SW60TI rotor and centrifuged at 30,000 g for 16 h at 4°C. For ADA gp140 a third purification step was necessary to get purified trimers. An anion-exchange chromatography on a HiTrap™ DEAE FF column was run with 0.5 ml/min flow rate and 0.5 ml fraction size. After each purification step, target fractions were concentrated and rebuffered with size exclusion filters (Amicon, 100 kDa cut off) in an appropriate buffer.

### ELISA

ELISA plates (high binding; Greiner) were coated over night at 4°C with purified trimeric gp140 proteins, monomeric gp120 proteins or BSA as negative control (100 ng per well). For analysis of CD4 induced epitopes by CG10 or 17b, 100 ng gp140 proteins and BSA were preincubated with 20 ng soluble 2 domain CD4 (Sino Biological Inc.) for 30 min at 37°C before coating. Plates were blocked after the first incubation step with 1% BSA (Serva) in PBS (Lonza) for at least 1 h at room temperature. For detection, monoclonal antibodies D19 (from Patricia Earl), CG10 (from Jon Gershoni), 17b, VRC01, VRC03, b13, F105, Md-1 (NIH), 447-52D, b12, 2F5, 246-D (from Polymun) as well as CD4-Fc (Abcam) were applied in 8 different concentrations between 0 and 10 nM or in different dilutions as indicated. α-p24 serum dilutions were used as negative control. Antibodies were incubated in PBS/0.1% Tween/0.1% BSA (B-PBS-T) for 1.5 h at room temperature. HRP-labeled α-human IgG or α-mouse IgG (for CG10 and α-p24) secondary antibodies were used 1:5,000 for 1 h at room temperature in B-PBS-T followed by chemiluminescence imaging. Between all incubations, the plates were washed three times with 300 μl PBS/0.1% Tween per well. Nonlinear regression fits were applied to the binding curves and K_D_ values were derived if applicable (PRISM software).

### Surface plasmon resonance (SPR) spectroscopy

Antibody binding experiments were performed on a ProteOn XPR36 system (Bio-Rad) at a constant temperature of 23°C. The evaluation was done with ProteOn Manager™ 3.1 software. As ligand, mAb 447-52D (200 μl, 0.015 mg/ml, pH 4.5) was immobilized on a ProteOn™ GLC Sensor Chip (Bio-Rad). As analytes, the gp140 proteins were injected in different concentrations from 0–200 nM (100 μl each). The association time was set to 60 s, the dissociation time to 300 s and the flow rate was 100 μl/min. The measured sensorgrams were fitted with a Langmuir binding model.

### Electron microscopy, 3D reconstruction and visualization

Samples were diluted to the final concentration (~0.02 mg/ml) and negatively stained on previously glow discharged (25 mA for 30 s) continuous carbon 200 mesh grids (Science service, Munich) using 2% uranyl formate. Grids were then transferred to a Tecnai12 transmission electron microscope operating a LaB_6_ electron source at 120 kV. Images were acquired at a nominal magnification of 71540× using a 4k×4k (bin 2) CCD camera (TVIPS, Munich) with a final resolution of 4.36 Å/pixel.

Image processing was performed on a 358 core AMD Opteron computer cluster. Particles were selected using the Appion [63] manual picker and Gaussian noise balls were used as starting models for half datasets. The half datasets were independently refined until no further improvement was seen using EMAN [44] and only combined for the last model building. As particles from the expected size were purified C3 symmetry was applied. During the refinement a spherical mask was applied and a final angular increment of 5° was used. The final 3D density maps were filtered to their resolution as determined by the FSC_1/2-bit_ criterion (25 Å) (for further details and references, see [64]). The threshold was approximately mass-corrected (assuming 3 × 140 = 420 kDa) for displaying. Visualization of density maps and the subsequent structural analysis, such as docking of the X-ray structure, was done using UCSF Chimera [65].

## Ethics statement

Research was carried out without any primary human or animal material and only standard HIV isolates were used that can be obtained through the NIH AIDS Research and Reference Program.

## Availability of supporting data

The data sets supporting the results of this article are available in the EMData Bank repository, EMDB 2657 [43] and 2659 [44].

## Competing interests

The authors declare that they have no competing interests.

## Authors’ contributions

PA, RD and UD designed research; PA, PH, SW, TD, VG, PB and RD performed the experiments; PA, JM, RT and RD analyzed data; RD, PA and UD wrote the manuscript. All authors read and approved the final manuscript.

## Authors’ information

Shared first authors: Philipp Arnold, Patricia Himmels and Svenja Weiß, Shared last authors: Ursula Dietrich and Ralf Dürr

## Supplementary Material

Additional file 1 Amino acid alignment of NL4-3 and NL4-3/ADA gp140 with indicated relevant epitopes. Click here for file

Additional file 2 CD4 and CD4bs mAb epitopes in NL4-3 and NL4-3/ADA gp140 constructs. Click here for file

Additional file 3 Purification of NL4-3 and NL4-3/ADA trimers and Western Blot of purified ADA gp140 proteins. Click here for file

Additional file 4 Antibody binding to gp140 constructs in ELISA experiments. Click here for file

Additional file 5 Dissociation constants derived from the ELISA antibody binding experiments of the gp140 constructs. Click here for file

Additional file 6 SPR binding and dissociation kinetics of the gp140 constructs to 447-52D mAb. Click here for file

Additional file 7 Single particles from NL4-3 and NL4-3/ADA gp140 electron micrographs. Click here for file

Additional file 8 Localization of N-Glycosylation sites in the NL4-3 gp140 density map. Click here for file

Additional file 9 Movie of the molecular model of NL4-3 gp140. Click here for file

Additional file 10 Superposition of fitted gp120 X-ray structures from NL4-3 and NL4-3/ADA gp140 models. Click here for file

Additional file 11 Comparison of our X4 NL4-3 and NL4-3/ADA gp140 with published CD4 independent SIV and R5 HIV-1 Env structures. Click here for file

Additional file 12 NL4-3 and NL4-3/ADA gp140 density maps at different thresholds. Click here for file

Additional file 13 Additional information: Supplemental methods and legends to supplemental Figures, Tables and Video (Additional files 1 - 12). Click here for file
