# Supplementary figures and images for: Antigenic and 3D structural characterization of soluble X4 and hybrid X4-R5 HIV-1 Env trimers
Source: Retrovirology. 2014 May 30;11:42. doi: 10.1186/1742-4690-11-42 (PMC4048260; doi:10.1186/1742-4690-11-42)

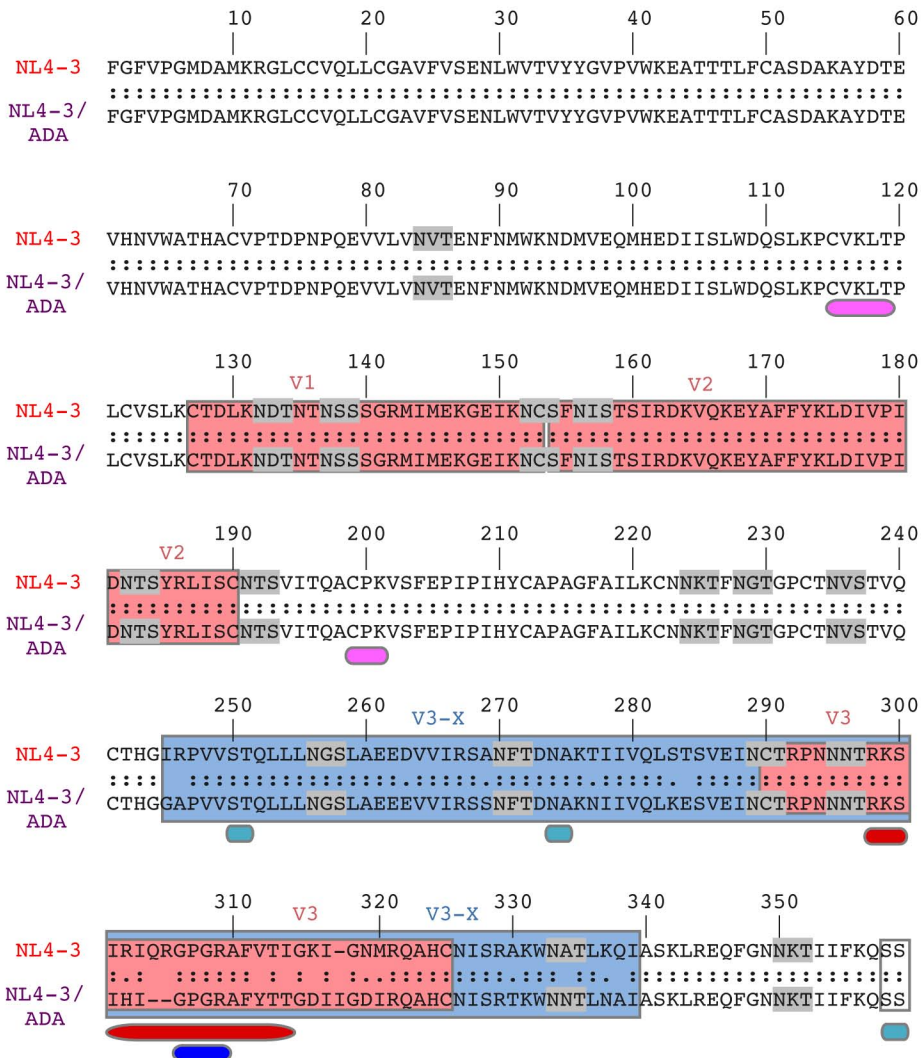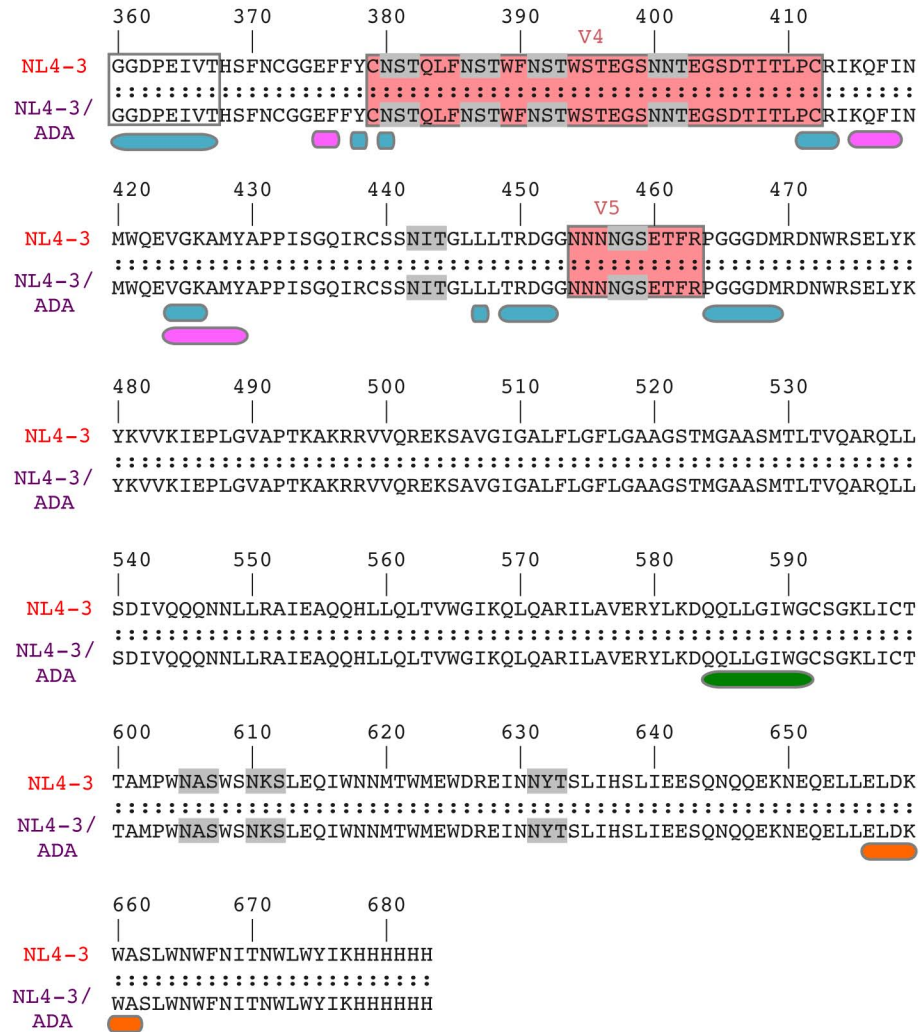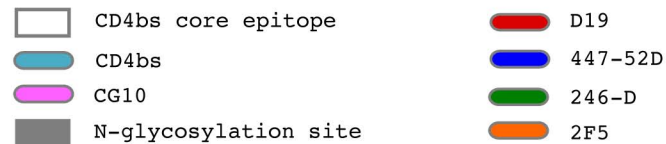

Supplement: Additional file 1 — Amino acid alignment of NL4-3 and NL4-3/ADA gp140 with indicated relevant epitopes. [file 1742-4690-11-42-S1.pdf]

*M* / kDa

NL4-3

*M* / kDa

NL4-3/ADA

*M* / kDa

ADA

300  
250  
170  
140  
100  
70  
55

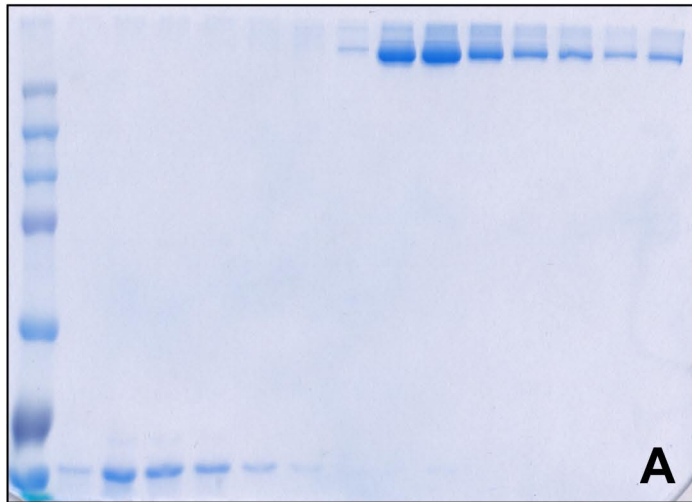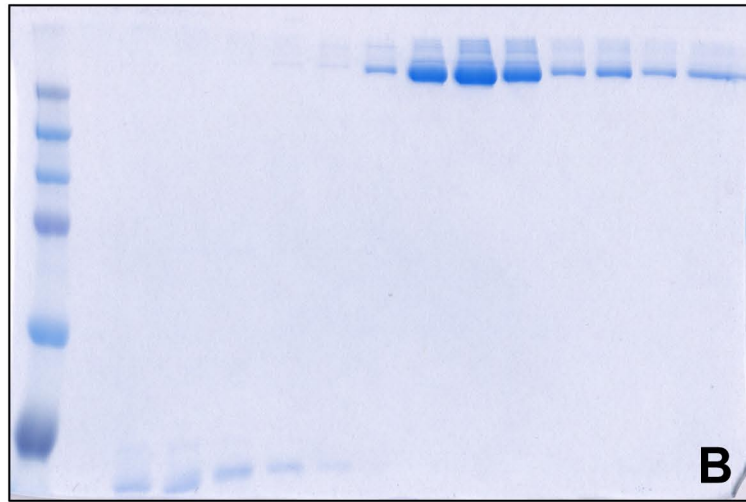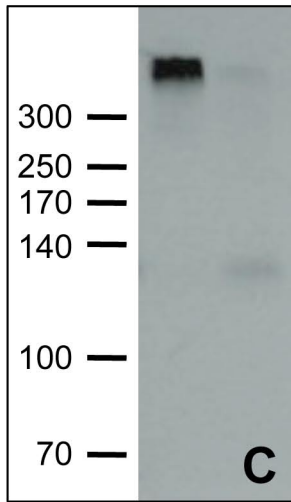

DTT: - +

Supplement: Additional file 3 — Purification of NL4-3 and NL4-3/ADA trimers and Western Blot of purified ADA gp140 proteins. [file 1742-4690-11-42-S3.pdf]

CD4i

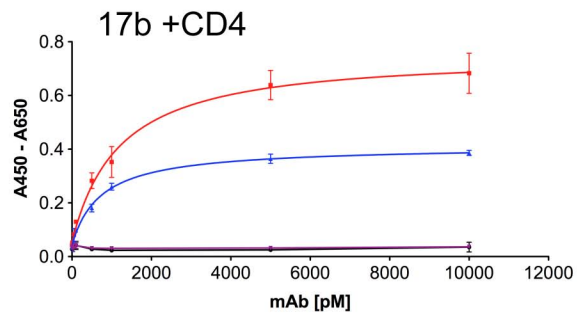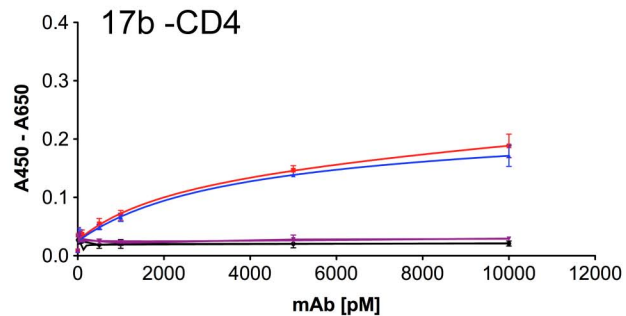

● BSA  
■ NL4-3  
▲ ADA  
▼ NL4-3/ADA

gp41

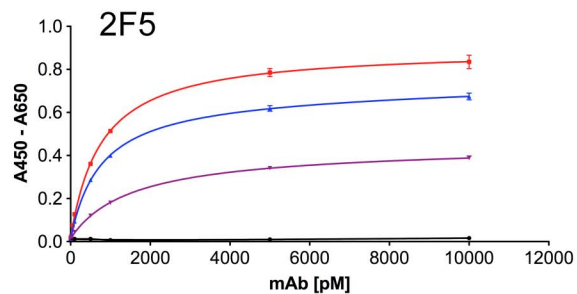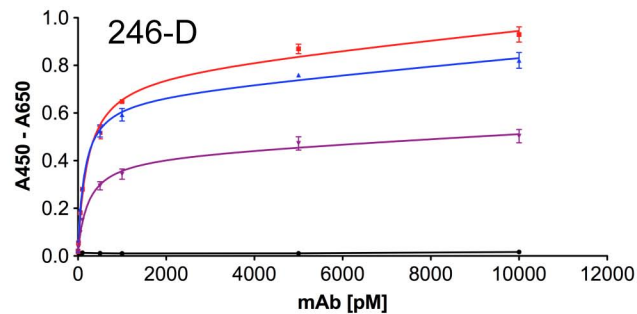

neg. control

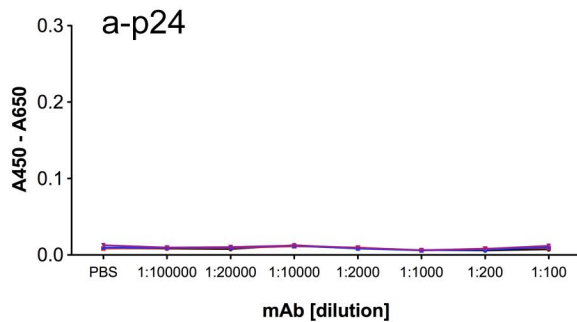

Supplement: Additional file 4 — Antibody binding to gp140 constructs in ELISA experiments. [file 1742-4690-11-42-S4.pdf]

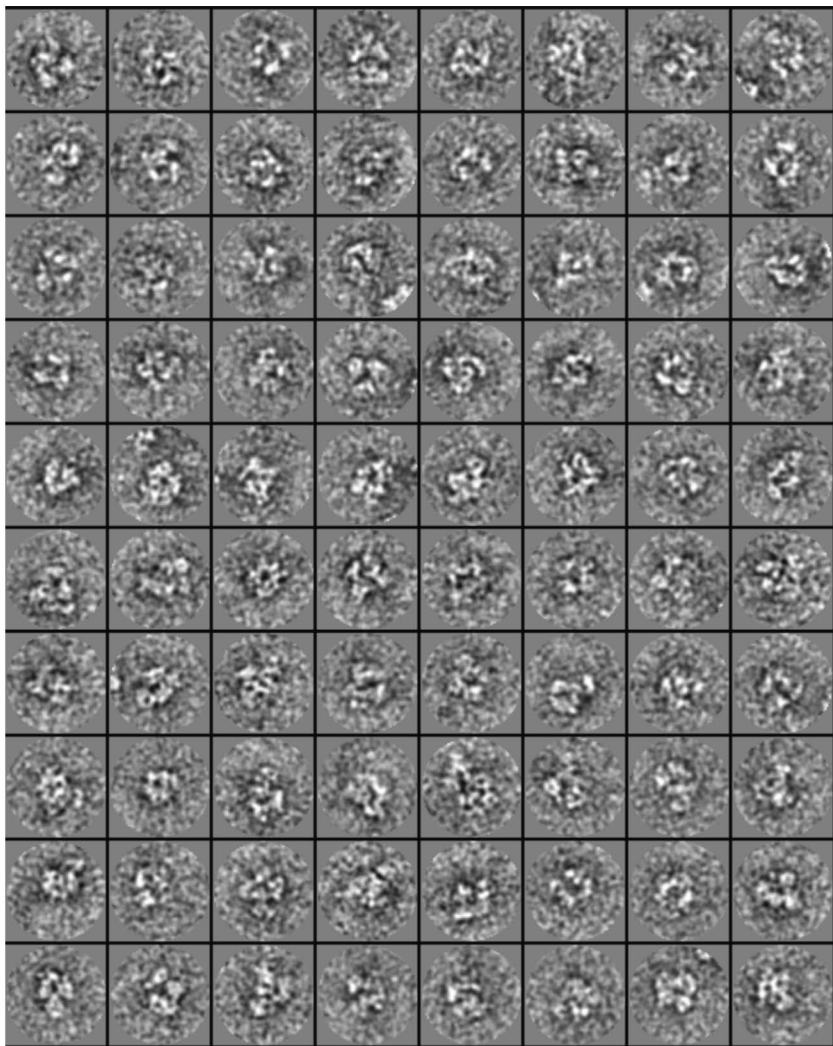

Single particles NL4-3 gp140

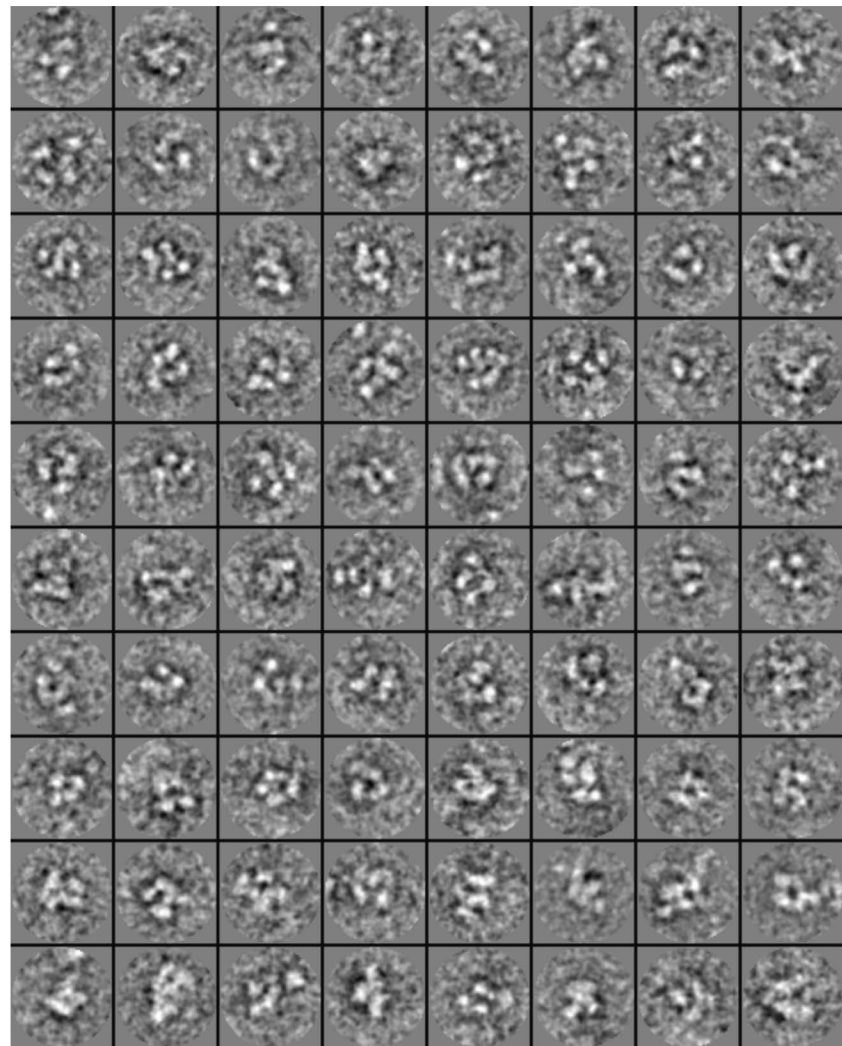

Single particles NL4-3/ADA

Supplement: Additional file 7 — Single particles from NL4-3 and NL4-3/ADA gp140 electron micrographs. [file 1742-4690-11-42-S7.pdf]

**A**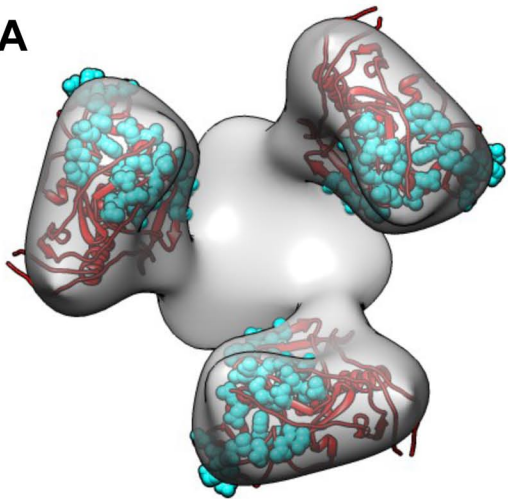**B**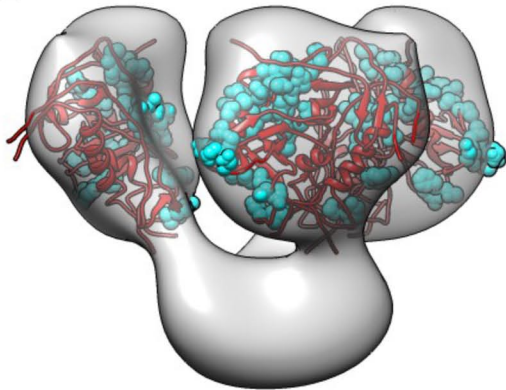

Supplement: Additional file 8 — Localization of N-Glycosylation sites in the NL4-3 gp140 density map. [file 1742-4690-11-42-S8.pdf]

**A**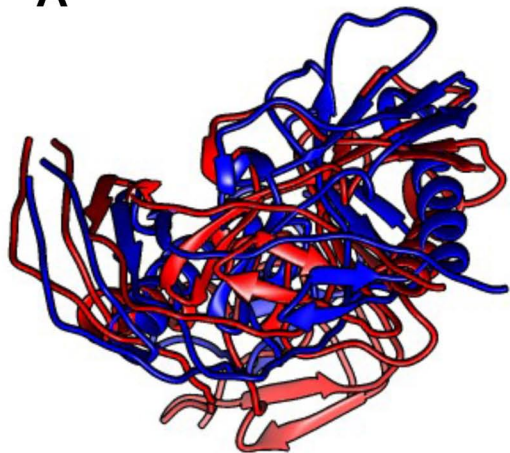**B**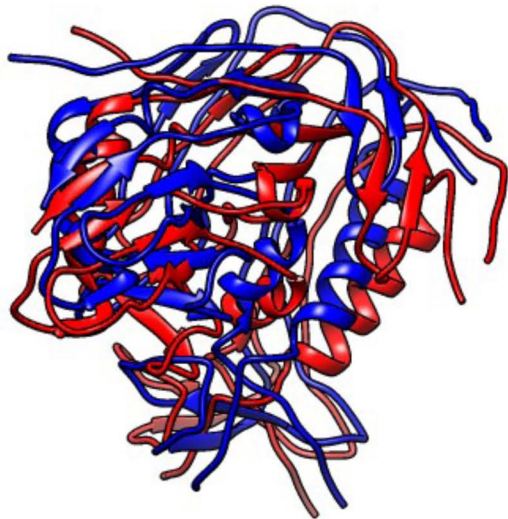

Supplement: Additional file 10 — Superposition of fitted gp120 X-ray structures from NL4-3 and NL4-3/ADA gp140 models. [file 1742-4690-11-42-S10.pdf]

**A**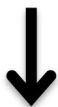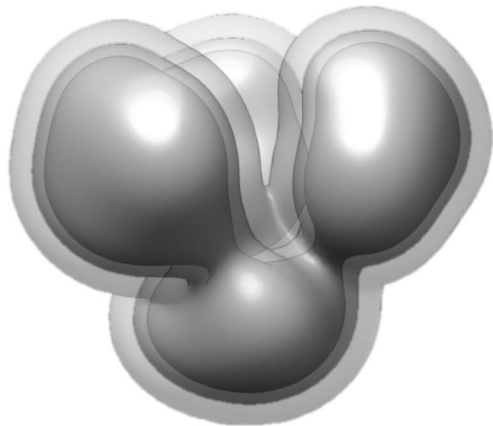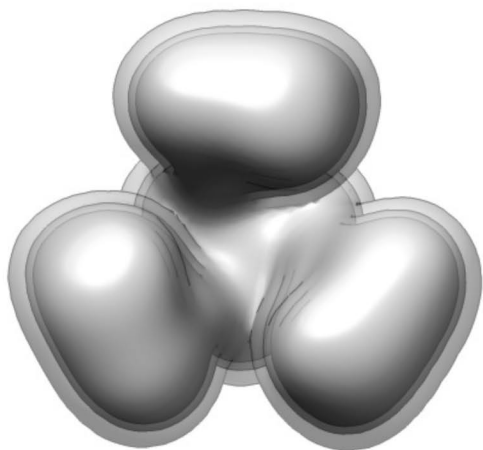**B**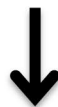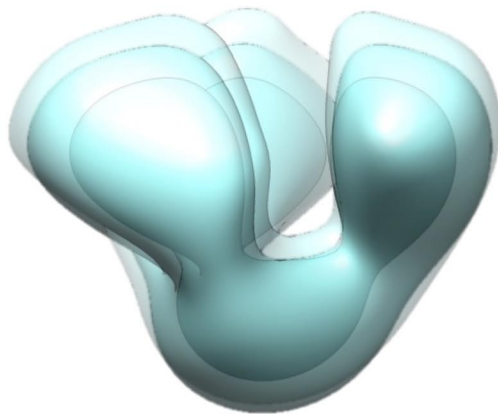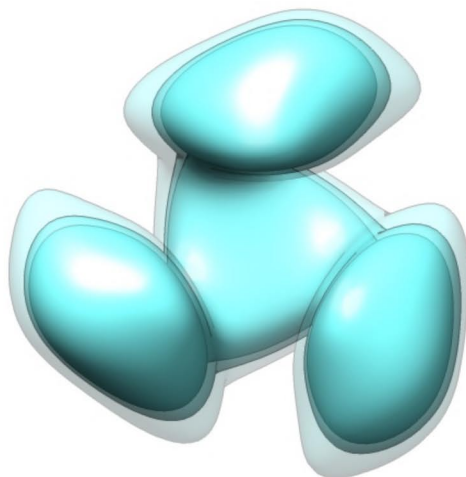

Supplement: Additional file 12 — NL4-3 and NL4-3/ADA gp140 density maps at different thresholds. [file 1742-4690-11-42-S12.pdf]
