# Supplementary material for: Antigenic and 3D structural characterization of soluble X4 and hybrid X4-R5 HIV-1 Env trimers
Source: Retrovirology. 2014 May 30;11:42. doi: 10.1186/1742-4690-11-42 (PMC4048260; doi:10.1186/1742-4690-11-42)
Supplement: Additional file 2 — CD4 and CD4bs mAb epitopes in NL4-3 and NL4-3/ADA gp140 constructs. [file 1742-4690-11-42-S2.pdf]

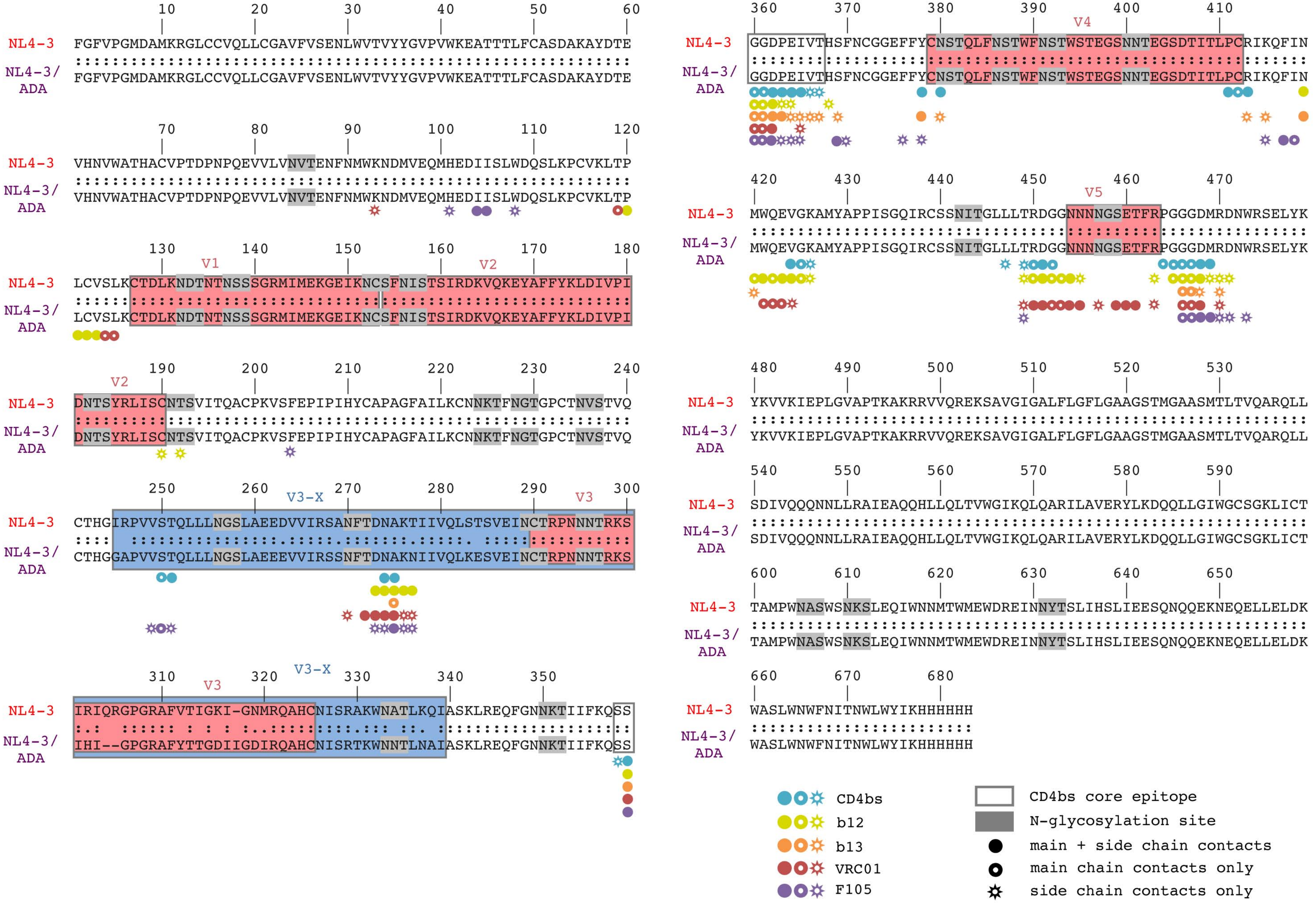

NL4-3 FGFVPGMDAMKRGLCCVQLLCGAVFVSENLWVTVYYGVPVWKEATTTLFCASDAKAYDTE
NL4-3/ADA FGFVPGMDAMKRGLCCVQLLCGAVFVSENLWVTVYYGVPVWKEATTTLFCASDAKAYDTE
NL4-3 VHNVWATHACVPTDPNPQEVVLVNVTENFNMWKNDMVEQMHEDIISLWDQSLKPCVKLTP
NL4-3/ADA VHNVWATHACVPTDPNPQEVVLVNVTENFNMWKNDMVEQMHEDIISLWDQSLKPCVKLTP
V1
V2
NL4-3 LCVSLKCTDLKNDTNTNSSSGRMIMEKGEIKNCSFNISTSIRDKVQKEYAFFYKLDIVPI
NL4-3/ADA LCVSLKCTDLKNDTNTNSSSGRMIMEKGEIKNCSFNISTSIRDKVQKEYAFFYKLDIVPI
V2
NL4-3 DNTSYRLISCNTSVITQACPKVSFEPIPIHYCAPAGFAILKCNNKTFNGTGPCTNVSTVQ
NL4-3/ADA DNTSYRLISCNTSVITQACPKVSFEPIPIHYCAPAGFAILKCNNKTFNGTGPCTNVSTVQ
V3-X
V3
NL4-3 CTHGIRPVVSTQLLLNGSLAEEDVVIRSANFTDNAKTIIVQLSTSVEINCTRPNNNTRKS
NL4-3/ADA CTHGGAPVVSTQLLLNGSLAEEEVVIRSSNFTDNAKNIIVQLKESVEINCTRPNNNTRKS
V3
V3-X
NL4-3 IRIQRGPGRAFVTIGKI-GNMRQAHCNISRAKWNATLKQIASKLREQFGNNKTIIFKQSS
NL4-3/ADA IHI--GPGRAFYTTGDIIGDIRQAHCNISRTKWNNTLNAIASKLREQFGNNKTIIFKQSS
V4
NL4-3 GGDPEIVTHSFNCGGEFFYCNSTQLFNSTWFNSTWSTEGSNNTEGSDTITLPCRIKQFIN
NL4-3/ADA GGDPEIVTHSFNCGGEFFYCNSTQLFNSTWFNSTWSTEGSNNTEGSDTITLPCRIKQFIN
V5
NL4-3 MWQEVGKAMYAPPISGQIRCSSNITGLLLTRDGGNNNNGSETFRPGGGDMRDNWRSELYK
NL4-3/ADA MWQEVGKAMYAPPISGQIRCSSNITGLLLTRDGGNNNNGSETFRPGGGDMRDNWRSELYK
NL4-3 YKVVKIEPLGVAPTKAKRRVVQREKSAVGIGALFLGFLGAAGSTMGAASMTLTVQARQLL
NL4-3/ADA YKVVKIEPLGVAPTKAKRRVVQREKSAVGIGALFLGFLGAAGSTMGAASMTLTVQARQLL
NL4-3 SDIVQQQNNLLRAIEAQQHLLQLTVWGIKQLQARILAVERYLKDQQLLGIWGCSGKLICT
NL4-3/ADA SDIVQQQNNLLRAIEAQQHLLQLTVWGIKQLQARILAVERYLKDQQLLGIWGCSGKLICT
NL4-3 TAMPWNASWSNKSLEQIWNNMTWMEWDREINNYTSLIHSLIEESQNQQEKNEQELLELDK
NL4-3/ADA TAMPWNASWSNKSLEQIWNNMTWMEWDREINNYTSLIHSLIEESQNQQEKNEQELLELDK
NL4-3 WASLWNWFNITNWLWYIKHHHHHH
NL4-3/ADA WASLWNWFNITNWLWYIKHHHHHH
CD4bs
b12
b13
VRC01
F105
CD4bs core epitope
N-glycosylation site
main + side chain contacts
main chain contacts only
side chain contacts only
