# Supplementary material for: Antigenic and 3D structural characterization of soluble X4 and hybrid X4-R5 HIV-1 Env trimers
Source: Retrovirology. 2014 May 30;11:42. doi: 10.1186/1742-4690-11-42 (PMC4048260; doi:10.1186/1742-4690-11-42)
Supplement: Additional file 11 — Comparison of our X4 NL4-3 and NL4-3/ADA gp140 with published CD4 independent SIV and R5 HIV-1 Env structures. [file 1742-4690-11-42-S11.pdf]

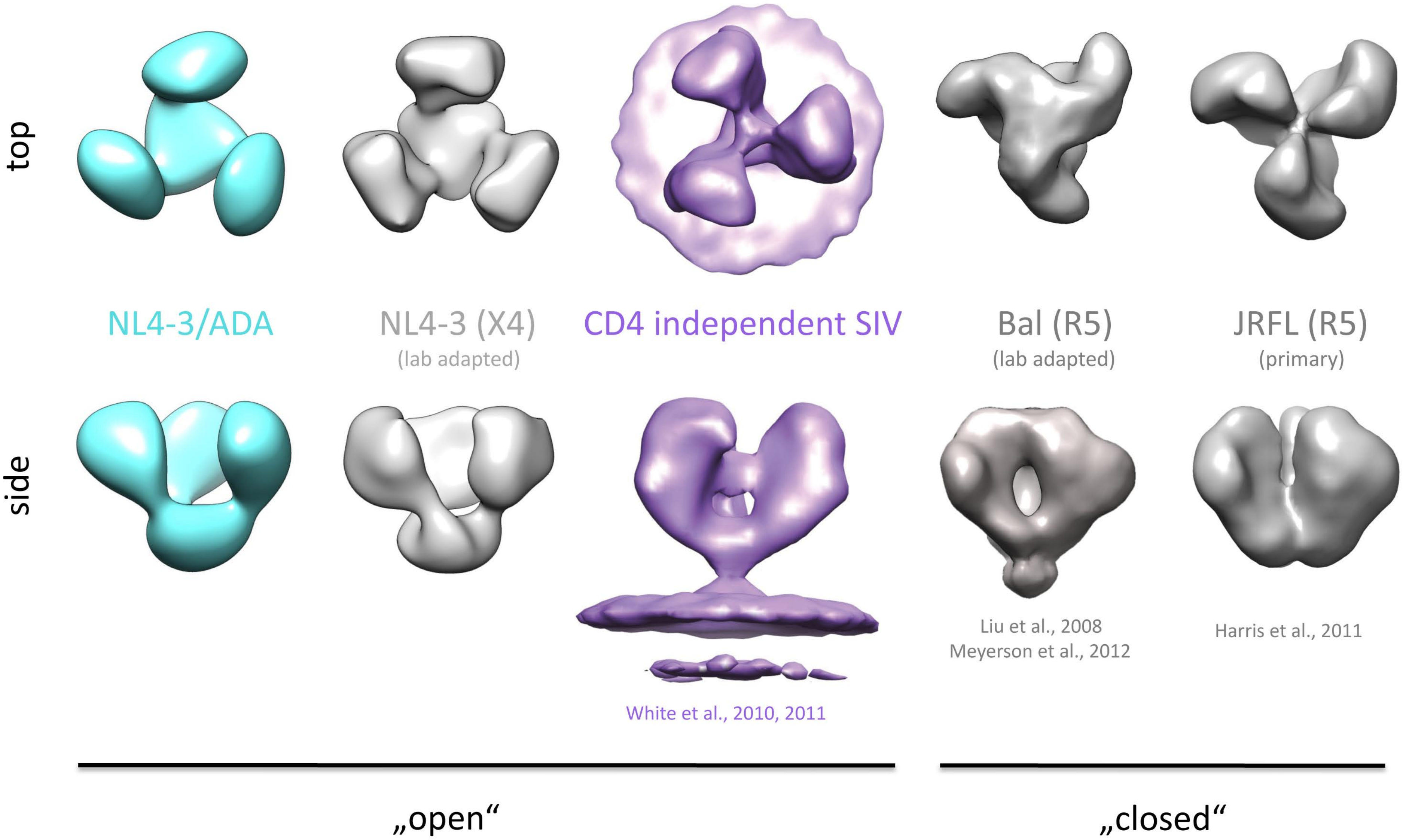
top
side
NL4-3/ADA
NL4-3 (X4)
(lab adapted)
CD4 independent SIV
Bal (R5)
(lab adapted)
JRFL (R5)
(primary)
White et al., 2010, 2011
Liu et al., 2008
Meyerson et al., 2012
Harris et al., 2011
„open“
„closed“
