# Supplementary material for: Antigenic and 3D structural characterization of soluble X4 and hybrid X4-R5 HIV-1 Env trimers
Source: Retrovirology. 2014 May 30;11:42. doi: 10.1186/1742-4690-11-42 (PMC4048260; doi:10.1186/1742-4690-11-42)
Supplement: Additional file 13 — Additional information: Supplemental methods and legends to supplemental Figures, Tables and Video (Additional files 1 - 12). [file 1742-4690-11-42-S13.docx]

**Additional information**

**Supplemental methods**

Cloning and expression of NL4-3 and NL4-3/ADA gp140

The NL4-3 gp140 sequence was amplified with the Phusion polymerase (NEB) from an NL4-3 full length clone (kindly provided by Matthias Dittmar) using a forward primer containing the tPA leader sequence and the start of the NL4-3 env sequence (5´-CGTACCCGGGATGGATGCAATGAAGAGAGGGCTCTGCTGTGTGCTGCTGCTGTGTGGAGCAGTCTTCGTTTCGGAAAATTTGTCGGTCACAGTTTATTAT-3´) and a reverse primer containing sequences of the C-terminus of NL4-3 gp140, a 6x-His tag, a stop codon after Lys648 and an EcoRI restriction site (5´-CGTAGGTNACCTGATCAGAATT CTCATCAGTGATGGTGATGGTGATGTTTTATATACCACAGCCA-3´). The construct was ligated into the pCR2.1 vector (Invitrogen) and the protease cleavage site was deleted by mutation of Arg476 into Ser476 (numbering concerning NL4-3 gp160) with the QuickChange site-directed mutagenesis kit and Pfu polymerase (Stratagene) (fwd: 5´-gtggtgcagagagaaaaGagcgcagtgggaacgataggag´3 and rev: 5´-ctcctatTcccactgcgctCttttctctctgcaccac-3´). The plasmid was digested with EcoRI and the excised gp140 fragment was subcloned into the EcoRI site of the pEE12.4 vector (Lonza). Transfection of purified plasmids was performed with Nanofectin (PAA) in CHO-K1 or CHO-Lec1 cells and transfected cells were selected with 25 µM methionine sulfoximine in GMEM culture media (Sigma) or alpha MEM media (PAN), respectively.

The hybrid NL4-3/ADA was generated with the NL4-3 gp140 pEE12.4 plasmid as template for the amplification of a PCR construct of the whole plasmid except the V3 spanning region to be exchanged. Terminally, unique restriction sites (AsiSI and AscI) were added with the primer AsiSI_for_NL4-3 (5’-GGGGCGATCGCTAGCAAATTAAGAGAACAATTTGGA-3’) and AscI_rev_NL4-3 (5’-TAATGGCGCGCCTCCATGTGTACATTGTACTGT-3’). For the insert, the V3 spanning region was amplified from the ADA-pSV Env plasmid (kindly provided by Dr. Rolf Kaiser, Cologne) and AsiSI and AscI restriction sites were terminally added with the primer AscI_for_ADA (5’-ATTGGCGCGCCAGTAGTGTCAACTCAA CTGCTG-3’) and AsiSI_rev_ADA (5’-CCCAGCGATCGCATTTAAAGTGTTATTCCATTT-3’). PCR constructs were digested with AsiSI and AscI, circularized by ligation and clones were analyzed for integrity of correct hybrid sequence.

SDS-PAGE and Western Blot

Proteins separated on 7.5% SDS-PAGE gels were either stained with Instant blue (Expedeon) or electro-transferred to Protran nitrocellulose membranes (Schleicher & Schuell) in a semi-dry transfer cell (BioRad). Membranes were blocked with 5% skim milk powder in PBS (Lonza) (MPBS). Detection was performed with mAb 447-52D (1:2,000) and HRP-labeled α-human IgG (goat) (1:5,000) in 0.1% MPBS with 0.1% Tween followed by chemiluminescence imaging. Between the incubation steps, blots were washed three times with 0.1% Tween in PBS. ProSieve Quad Color Protein Marker (Biozym) was used as protein standard.

Alignment

Pairwise protein sequence alignment was performed using LALIGN (EMBL-EBI).

**Legends to supplemental figures, tables and video**

**Additional file 1. Amino acid alignment of NL4-3 and NL4-3/ADA gp140 with indicated relevant epitopes.**

Amino acid sequence alignment of NL4-3 and NL4-3/ADA gp140 constructs with variable regions colored and labeled in red and the exchange of the extended V3 region colored and labeled in blue. Antibody epitopes and N-glycosylation sites as well as the CD4 binding site are marked as indicated in the legend. CG10 epitopes were chosen as representatives for CD4i coreceptor binding site mAbs (17b, CG10). CD4bs mAb epitopes are illustrated in detail in Additional file 2. The discontinous gp41 Md-1 epitope [[1](#_ENREF_1)], which is located between aa 557 to 666 (numbering of our NL4-3 gp140 construct) is omitted in the scheme.

**Additional file 2. CD4 and CD4bs mAb epitopes in NL4-3 and NL4-3/ADA gp140 constructs.**

Amino acid sequence alignment of NL4-3 and NL4-3/ADA gp140, as shown in Additional file 1, with marked epitopes for CD4 as well as mAbs b12, b13, F105 [[2](#_ENREF_2)] and VRC01 [[3](#_ENREF_3)], including data about main-chain and/or side-chain contacts.

**Additional file 3. Purification of NL4-3 and NL4-3/ADA trimers and Western Blot of purified ADA gp140 proteins.**

Lectin purification with subsequent glycerol gradient purification, as shown in (**A**) for the NL4-3 and in (**B**) for the NL4-3/ADA construct yielded highly purified gp140 trimers. (**C**) Western Blot analysis of purified ADA gp140 trimers after lectin, size exclusion, and anion exchange chromatography under non-reducing and reducing conditions (50 mM DTT).

**Additional file 4. Antibody binding to gp140 constructs in ELISA experiments.**

The antigenic profiles of the gp140 constructs NL4-3 (X4), ADA (R5) and the hybrid NL4-3/ADA were evaluated in ELISA experiments with selected monoclonal antibodies (mAbs) against CD4-induced epitopes at the coreceptor binding site (17b) (with and without prior CD4 activation) [[4](#_ENREF_4)], the gp41 loop (246-D) [[5](#_ENREF_5)] and the gp41 membrane proximal external region (2F5) [[6](#_ENREF_6)]. Polyclonal serum against the HIV capsid protein p24 was used as a negative control. Where applicable, nonlinear regression fits are shown in the diagrams instead of connected data points. K_D_ values were derived from the nonlinear regression fits (PRISM software) and listed in the table of Additional file 4. The data is representative for at least three replicate experiments with each data point of the binding curves determined in triplicates with indicated mAb concentrations. Error bars indicate the standard deviation.

**Additional file 5. Dissociation constants derived from the ELISA antibody binding experiments of the gp140 constructs.**

Nonlinear regression fits were deduced from the antibody binding curves of the NL4-3, ADA, and NL4-3/ADA gp140 constructs. K_D_ values were calculated with PRISM software. The mean dissociation constants (K_D_) were calculated from the results of at least 3 replicate ELISA experiments and the respective standard deviations (SD) are indicated in [pM], or for the unpurified mAb Md-1 ^(#)^ in [x * 1/1000] (n.a.: not applicable).

**Additional file 6. SPR binding and dissociation kinetics of the gp140 constructs to 447-52D mAb.**

The mean dissociation constants (K_D_ in [M]), association rates (k_on_ in [1/Ms]) and dissociation rates (k_off_ in [1/s]) from 3 replicate SPR experiments (Biorad ProteOn XPR36 system) and the respective standard deviations (SD) are indicated.

**Additional file 7. Single particles from NL4-3 and NL4-3/ADA gp140 electron micrographs.**

Random selection of 80 picked single particles of NL4-3 and NL4-3/ADA trimers prior classification.

**Additional file 8. Localization of N-glycosylation sites in the NL4-3 gp140 density map.**

Top view (**A**) and side view (**B**) of the NL4-3 reconstruction with fitted gp120 X-ray structure (red) and the NXT/S sites marked by light blue spheres. The glycosylation sites mainly face outwards, further assessing the fidelity of our fittings.

**Additional file 9. Movie of the molecular model of NL4-3 gp140.**

An animated version of the NL4-3 gp140 model, as in Figure 6 bottom row, is shown here to better visualize the topology of the gp120 fitting and the illustrated Env substructures in the density maps.

**Additional file 10. Superposition of fitted gp120 X-ray structures from NL4-3 and NL4-3/ADA gp140 models.**

Superposition of the fitted gp120 X-ray structures from the NL4-3 (red) and NL4-3/ADA (blue) models in top view (**A**) and side view (**B**). Matching was performed with gp120 models after stable docking of the respective density maps. Note that both molecular models almost superimpose.

**Additional file 11. Comparison of our X4 NL4-3 and NL4-3/ADA gp140 with published CD4 independent SIV and R5 HIV-1 Env structures.**

Comparison of our NL4-3/ADA (turquoise) and NL4-3 (grey) gp140 reconstructions with a CD4 independent SIV Env (EMDB# 5274) [[7](#_ENREF_7)], a laboratory-adapted HIV-1 R5 Env (Bal) (EMDB# 5019) [[8](#_ENREF_8)] and a primary HIV-1 R5 Env (JRFL) (EMDB # 1930) [[9](#_ENREF_9)]. Note that all density maps exhibit a propeller-like Env architecture, however with different degrees of "openness".

**Additional file 12. NL4-3 and NL4-3/ADA gp140 density maps at different thresholds.** 3D-reconstruction of NL4-3 (A) and NL4-3/ADA (B) are shown in different thresholds in top view and in side view. The solid small structure displays the mass correlated threshold. Even at higher thresholds there is a clear separation between the three masses at the top of the reconstructions (arrow). This points towards a more open Env conformation compared to previously published Env trimers derived from R5 tropic HIV stains that show a much closer arrangement of the three masses at the top (compare Additional file 11).

**Additional References**

1. Myers R, Meiller T, Falkler J, W., Patel J, Joseph J: **A Human Monoclonal Antibody to a Cryptic gp41 Epitope on HIV-1 Infected Cells.** *Abstr Gen Meet Am Soc Microbiol* 1993, **93:444**.

2. Chen L, Kwon YD, Zhou T, Wu X, O'Dell S, Cavacini L, Hessell AJ, Pancera M, Tang M, Xu L, Yang ZY, Zhang MY, Arthos J, Burton DR, Dimitrov DS, Nabel GJ, Posner MR, Sodroski J, Wyatt R, Mascola JR, Kwong PD: **Structural basis of immune evasion at the site of CD4 attachment on HIV-1 gp120.** *Science* 2009, **326:**1123-1127.

3. Li Y, O'Dell S, Walker LM, Wu X, Guenaga J, Feng Y, Schmidt SD, McKee K, Louder MK, Ledgerwood JE, Graham BS, Haynes BF, Burton DR, Wyatt RT, Mascola JR: **Mechanism of neutralization by the broadly neutralizing HIV-1 monoclonal antibody VRC01.** *J Virol* 2011, **85:**8954-8967.

4. Robinson JE, Holton D, Pacheco-Morell S, Liu J, McMurdo H: **Identification of conserved and variant epitopes of human immunodeficiency virus type 1 (HIV-1) gp120 by human monoclonal antibodies produced by EBV-transformed cell lines.** *AIDS research and human retroviruses* 1990, **6:**567-579.

5. Binley JM, Ditzel HJ, Barbas CF, 3rd, Sullivan N, Sodroski J, Parren PW, Burton DR: **Human antibody responses to HIV type 1 glycoprotein 41 cloned in phage display libraries suggest three major epitopes are recognized and give evidence for conserved antibody motifs in antigen binding.** *AIDS research and human retroviruses* 1996, **12:**911-924.

6. Muster T, Steindl F, Purtscher M, Trkola A, Klima A, Himmler G, Ruker F, Katinger H: **A conserved neutralizing epitope on gp41 of human immunodeficiency virus type 1.** *Journal of virology* 1993, **67:**6642-6647.

7. White TA, Bartesaghi A, Borgnia MJ, de la Cruz MJ, Nandwani R, Hoxie JA, Bess JW, Lifson JD, Milne JL, Subramaniam S: **Three-dimensional structures of soluble CD4-bound states of trimeric simian immunodeficiency virus envelope glycoproteins determined by using cryo-electron tomography.** *Journal of virology* 2011, **85:**12114-12123.

8. Liu J, Bartesaghi A, Borgnia MJ, Sapiro G, Subramaniam S: **Molecular architecture of native HIV-1 gp120 trimers.** *Nature* 2008, **455:**109-113.

9. Harris A, Borgnia MJ, Shi D, Bartesaghi A, He H, Pejchal R, Kang YK, Depetris R, Marozsan AJ, Sanders RW, Klasse PJ, Milne JL, Wilson IA, Olson WC, Moore JP, Subramaniam S: **Trimeric HIV-1 glycoprotein gp140 immunogens and native HIV-1 envelope glycoproteins display the same closed and open quaternary molecular architectures.** *Proc Natl Acad Sci U S A* 2011, **108:**11440-11445.
